# Assessment of H_2_S *in vivo* using the newly developed mitochondria-targeted mass spectrometry probe MitoA

**DOI:** 10.1074/jbc.M117.784678

**Published:** 2017-03-20

**Authors:** Sabine Arndt, Carlos D. Baeza-Garza, Angela Logan, Tiziana Rosa, Rudolf Wedmann, Tracy A. Prime, Jack L. Martin, Kourosh Saeb-Parsy, Thomas Krieg, Milos R. Filipovic, Richard C. Hartley, Michael P. Murphy

**Affiliations:** From the ‡ MRC Mitochondrial Biology Unit, University of Cambridge, Hills Road, Cambridge CB2 0XY, United Kingdom,; the § WestCHEM School of Chemistry, University of Glasgow, Glasgow G12 8QQ, United Kingdom,; the ¶ Department of Medicine, University of Cambridge, Biomedical Campus, Cambridge, CB2 2QQ, United Kingdom,; the ‖ Department of Chemistry and Pharmacy, Friedrich-Alexander University of Erlangen-Nuremberg, Egerlandstrasse,1, 91058 Erlangen, Germany,; the ‡‡ University of Bordeaux, IBGC, UMR 5095, F-33077 Bordeaux, France, and; the ** Department of Surgery and Cambridge NIHR Biomedical Research Centre, Biomedical Campus, University of Cambridge, Cambridge CB2 2QQ, United Kingdom

**Keywords:** chemical biology, hydrogen sulfide, hypoxia, mass spectrometry (MS), mitochondria, analytical chemistry, chemical biology, energy metabolism, mitochondria, hypoxia, hydrogen sulfide

## Abstract

Hydrogen sulfide (H_2_S) is produced endogenously *in vivo* and has multiple effects on signaling pathways and cell function. Mitochondria can be both an H_2_S source and sink, and many of the biological effects of H_2_S relate to its interactions with mitochondria. However, the significance of mitochondrial H_2_S is uncertain, in part due to the difficulty of assessing changes in its concentration *in vivo*. Although a number of fluorescent H_2_S probes have been developed these are best suited to cells in culture and cannot be used *in vivo*. To address this unmet need we have developed a mitochondria-targeted H_2_S probe, MitoA, which can be used to assess relative changes in mitochondrial H_2_S levels *in vivo*. MitoA comprises a lipophilic triphenylphosphonium (TPP) cation coupled to an aryl azide. The TPP cation leads to the accumulation of MitoA inside mitochondria within tissues *in vivo*. There, the aryl azido group reacts with H_2_S to form an aryl amine (MitoN). The extent of conversion of MitoA to MitoN thus gives an indication of the levels of mitochondrial H_2_S *in vivo*. Both compounds can be detected sensitively by liquid chromatography tandem mass spectrometry (LC-MS/MS) analysis of the tissues, and quantified relative to deuterated internal standards. Here we describe the synthesis and characterization of MitoA and show that it can be used to assess changes in mitochondrial H_2_S levels *in vivo*. As a proof of principle we used MitoA to show that H_2_S levels increase *in vivo* during myocardial ischemia.

## Introduction

There is considerable interest in the biological roles of hydrogen sulfide (H_2_S), both as an endogenously produced modulator of mammalian metabolism and for its potential biomedical effects when generated pharmacologically (1–3). Changes in endogenous H_2_S levels have been claimed to impact on a diverse range of physiological processes, including neuronal function, blood pressure, angiogenesis, oxygen sensing, inflammation, and mitochondrial energy production (4–16), and exposure to H_2_S can induce a suspended animation-like state in mice (17), but not in larger animals (18, 19).

**Figure 1.**
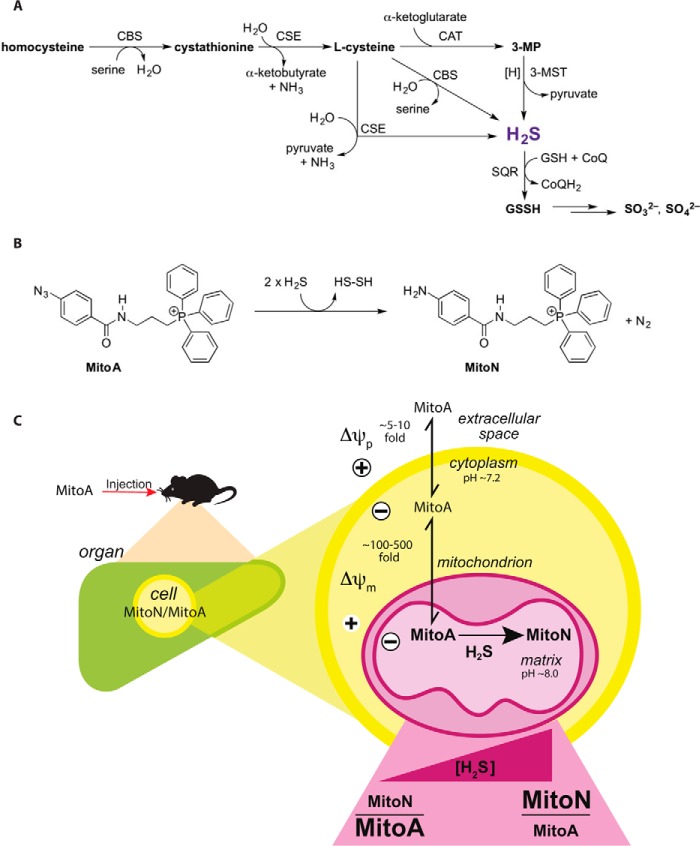
**Overview of endogenous H_2_S production and rationale for its detection by MitoA.** *A*, overview of H_2_S metabolism (sulfur-containing compounds in bold). *CBS*, cystathionine β-synthase; *CSE*, cystathionine β-lyase; *CAT*, cysteine aminotransferase; *GSSH*, glutathione persulfide; *3-MP*, 3-mercaptopyruvate; *3-MST*, 3-mercaptopyruvate sulfurtransferase. *B,* reaction of MitoA with H_2_S to form MitoN. *C,* model of uptake of MitoA into mitochondria *in vivo*, followed by its conversion to MitoN upon reaction with H_2_S. The subsequent extraction of MitoA and MitoN from the tissue and analysis by LC-MS/MS enables changes in the levels of H_2_S *in vivo* to be inferred.

Within mammals there are three main enzymatic sources of H_2_S: cystathionine β-synthase and cystathionine γ-lyase in the cytosol, and mitochondrial 3-mercaptopyruvate sulfurtransferase in conjunction with cysteine aminotransferase (Fig. 1*A*) (20). Once generated, H_2_S can be oxidized by sulfide:quinone oxidoreductase (SQR)^4^ in the mitochondrial inner membrane, passing electrons to the CoQ pool, and in parallel generating glutathione persulfide in the matrix, which is further metabolized to thiosulfate/sulfite/sulfate in the mitochondrial matrix and intermembrane space (21, 22) (Fig. 1*A*).

The biological activity of H_2_S arises from four interrelated processes: protein persulfidation, binding to metalloprotein centers (most notably, inhibition of cytochrome oxidase), interaction with NO signaling pathways, and as an antioxidant (14, 20). Protein persulfidation is a recently emerging mode of reversible posttranslational modification (PTM) of protein thiols in which H_2_S can react with a disulfide or a sulfenic acid, but not unmodified protein thiols, to form a persulfidated protein thiol (PrSSH), which can be reduced back to a thiol by the glutathione or thioredoxin systems (23, 24). This reversible PTM can in principle modify protein activity in a similar way to other PTMs, or can act as a releasable pool of H_2_S; however, the extent and physiological significance of protein persulfidation is still emerging. H_2_S can also inhibit cytochrome oxidase (25), which is why high concentrations of H_2_S are toxic, but whether these interactions with cytochrome oxidase or other heme proteins are of physiological or pharmacological importance is unclear. H_2_S signaling is intimately interwoven with that of NO, due to the formation of HSNO and other intermediates, and it is likely that protein modification by persulfidation and *S*-nitrosation are facets of a general signaling pathway (26, 27). Finally, H_2_S may also contribute to antioxidant defenses, by reacting directly with oxidants, or indirectly through its interactions with protein thiols (20, 28).

Abnormal H_2_S levels have been reported in a range of pathologies including Down syndrome (29), diabetes (30), liver cirrhosis, and in ethylmalonic acid encephalopathy, which occurs due to mutation to *ETHE1* (31). However, the factors that modify H_2_S levels *in vivo* under normal and pathological conditions are obscure. In addition, the contribution of H_2_S generation to the efficacy of pharmacological agents that release H_2_S is uncertain. Therefore, understanding how the levels of H_2_S change *in vivo* is vital to understanding its roles in physiology, pathology, and pharmacology.

There are a number of methods to assess H_2_S in cells using fluorescent probes that allow real-time detection of changes in H_2_S levels (32–40). Although these methods can be applied to the surfaces of animals (41), and to blood *ex vivo* (42), they cannot be used in whole animals *in vivo*. Consequently, there are considerable uncertainties about how H_2_S tissue levels change in response to physiological, pathological, or pharmacological events. These uncertainties are a major impediment to better understanding the biological roles of H_2_S and its downstream targets.

To overcome this obstacle, we have developed MitoA, a mitochondria-targeted mass spectrometry probe for H_2_S detection *in vivo* (Fig. 1*B*). The probe comprises an H_2_S-sensitive aryl azide moiety coupled to the lipophilic triphenylphosphonium (TPP) cation (Fig. 1*B*). The TPP cation targets a wide range of bioactive and probe compounds to mitochondria *in vivo* in response to the mitochondrial membrane potential (43, 44), and thus should lead to the rapid uptake of MitoA into mitochondria *in vivo*. Within the mitochondrial matrix the aryl azide moiety is intended to react with H_2_S to form the aryl amine, MitoN (Fig. 1*B*). This chemistry is based on that of widely used H_2_S-sensitive fluorescent probes, which rely on the reaction of a non-fluorescent aryl azide to give a fluorescent aryl amine (33, 37, 45, 46). The reaction occurs by initial nucleophilic attack of HS^−^ on the electrophilic terminal nitrogen of the aryl azide, followed by a rate-determining reaction with a second HS^−^, forming HS_2_^−^, N_2_, and the aryl amine (37) (Fig. 1*B*). Thus, MitoA can be injected into animals and the extent of generation of the exomarker MitoN will depend on the levels of H_2_S within mitochondria *in vivo*. Similar exomarker approaches have been used *in vivo* to assess hydrogen peroxide using MitoB (47, 48), and glyoxals through MitoG (49, 50). To analyze MitoA and its conversion to MitoN, both are extracted from animal tissue and then analyzed by LC-MS/MS relative to deuterated internal standards (Fig. 1*C*). Because MitoA is mainly present within mitochondria *in vivo*, it will report on the local concentration of H_2_S within mitochondria. However, because H_2_S diffuses rapidly across cellular membranes, H_2_S levels in the mitochondria are likely to reflect or influence overall changes in H_2_S within cells *in vivo*. Here we report the development of MitoA and show that it can be used to assess changes in H_2_S *in vivo*.

## Results and discussion

### Reaction of MitoA with H_2_S to form MitoN

**Figure 2.**
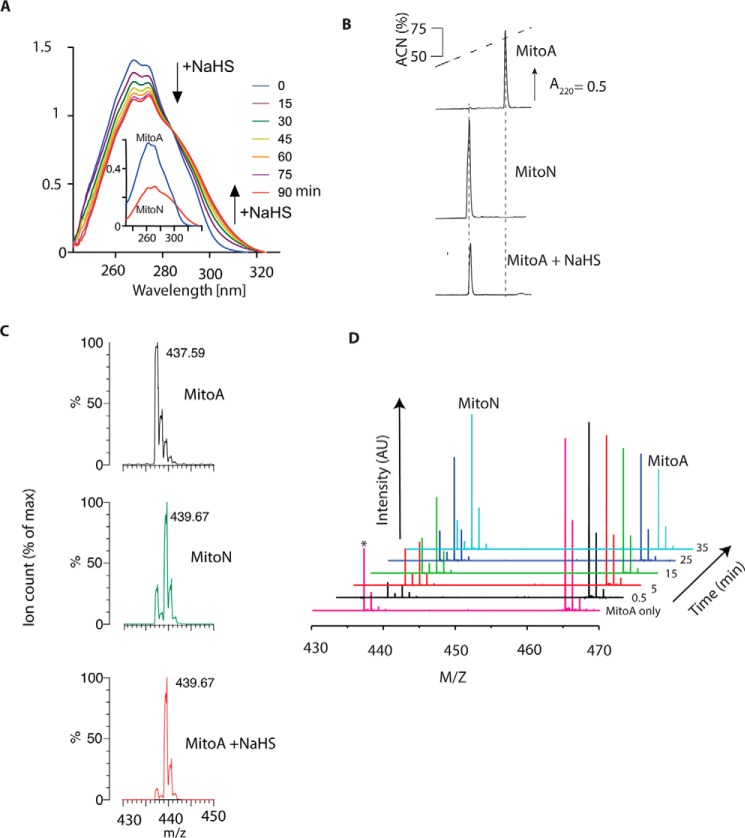
**Reaction of MitoA with H_2_S to form MitoN.** *A,* UV-visual absorbance spectra of MitoA reacting with NaHS. MitoA (50 μm) in KCl buffer was mixed with 5 mm NaHS and spectra were taken every 15 min. The *inset* shows spectra of pure MitoA and MitoN (25 μm each). *B*, RP-HPLC analysis of MitoA, MitoN, and its reactivity with H_2_S. Standards of 10 nmol of MitoA (*upper trace*) or MitoN (*middle trace*) were separated by RP-HPLC. For the *lower trace* MitoA (100 μm) in KCl buffer was incubated overnight with 1 mm NaHS at room temperature. Then 5 nmol eq of starting MitoA was analyzed by RP-HPLC. Spiking the NaHS-treated MitoA with MitoN increased the MitoN peak (data not shown). *C*, mass spectra of MitoA, MitoN, or MitoA incubated overnight with NaHS. Mass spectra were taken of MitoA (100 μm), MitoN (100 μm), or MitoA (100 μm) incubated with 1 mm NaHS in KCl buffer, pH 7.2, overnight at room temperature. Samples were diluted in 20% ACN, 0.1% FA and analyzed within 5 h. Predicted *m*/*z*: MitoA, [C_28_H_26_N_4_OP]^+^ = 465.18; nitrene from neutral loss of N_2_, [C_28_H_26_N_2_OP]^+^ = 437.17; MitoN, [C_28_H_29_N_2_OP]^+^ = 439.19. The MS of MitoA incubated at room temperature overnight was the same as that for freshly prepared MitoA (data not shown). *D,* time-resolved analysis of the reaction of MitoA with H_2_S. MitoA (100 μm) was mixed with 2.5 mm H_2_S in 300 mm ammonium carbonate, pH 7.4, and sprayed continuously over 45 min into an ultra high resolution mass spectrometer (maXis Bruker Daltonics). Spectra at selected time points are shown. *Asterisk* shows the nitrene formed from MitoA by the neutral loss of N_2_.

We first determined whether MitoA reacted with H_2_S to form MitoN by measuring the effect of H_2_S on the UV absorption spectra of MitoA over time (Fig. 2*A*). Addition of NaHS, which rapidly generates H_2_S (p*K_a_* = 6.8 (51)), led to the gradual conversion of MitoA to MitoN. RP-HPLC analysis of MitoA following overnight incubation with excess NaHS showed its complete conversion to MitoN (Fig. 2*B*). We could readily detect MitoA and MitoN by mass spectrometry (MS) and their spectra matched those predicted from the natural isotope distribution (supplemental Fig. S2). There was decomposition of MitoA ([C_28_H_26_N_4_OP]^+^; predicted *m*/*z* 465.18) by neutral loss of N_2_ to form the nitrene ([C_28_H_26_N_2_OP]^+^; predicted *m*/*z* 437.17), the extent of which depended on MS conditions (Fig. 2*C*). MS analysis of MitoA after overnight incubation with excess NaHS showed formation of MitoN ([C_28_H_29_N_2_OP]^+^; predicted *m*/*z* 439.19) further confirming that MitoA reacts with H_2_S to form only MitoN (Fig. 2*C*); under these MS conditions only the nitrene product of MitoA was found. When the reaction of MitoA with H_2_S was monitored continuously using time-resolved MS, where both MitoA and its nitrene product were observed, the gradual loss of MitoA and the accumulation of MitoN were seen over time, consistent with MitoA reacting selectively with H_2_S to generate MitoN (Fig. 2*D*).

The interaction of MitoA with other potential reactive species that it may encounter *in vivo* was assessed by RP-HPLC (Table 1). None of the tested compounds reacted to any significant extent with MitoA to form MitoN (Table 1): the greatest conversion of MitoA to MitoN was 7.9% and required incubation with 10 mm glutathione for 24 h. A significant potential confounder for the selectivity of MitoA for H_2_S is the possibility of its reacting with low molecular weight and protein persulfides. Therefore we next assessed the direct reactivity of MitoA with NAP-SSH, a low molecular weight persulfide derivative of *N*-acetylpenicillamine (NAP) (24, 52). A complication of analyzing persulfides is that they spontaneously release H_2_S, making it difficult to separate the reaction of MitoA with H_2_S from that with the persulfide itself (24). To address this, we compared the reaction of MitoA with NAP-SSH to that of MitoA with the same concentration of H_2_S (supplemental Fig. S3). These experiments showed the expected reaction of MitoA with H_2_S with formation of MitoN, whereas under the same conditions the reaction of MitoA with NAP-SSH to form MitoN was negligible (supplemental Fig. S3). Furthermore, the incubation of 5 μm persulfidated human serum albumin with 10 μm MitoA led to negligible (<7%) consumption of MitoA over 4 h at room temperature (data not shown), most probably due to spontaneous decay of persulfides to release H_2_S (53).

**Table 1:** **MitoA (10 μm) was incubated with each substance for the indicated time and temperature in KCl buffer and analyzed by RP-HPLC** Dimethyl trisulfide was dissolved in 70% EtOH. For NO, DetaNONOate (50 μm) was disolved in KCl buffer, which had been deoxygenated by bubbling with argon for 30 min. Superoxide was generated using 5 milliunits/ml of xanthine oxidase and 1 mm hypoxanthine in KCl buffer and its production quantified as the SOD-sensitive reduction of ferricytochrome *c*. Reactivity = (MitoN × 100)/(MitoN + MitoA).

	Concentration	Incubation condition	Reactivity
			%
H_2_S	100 μm	4 h, RT*^a^*	94
Glutathione	5 mm	24 h, 37 °C	7.3
	5 mm	4 h, 37 °C	2.2
	10 mm	24 h, 37 °C	7.9
	10 mm	4 h, 37 °C	4.6
Cysteine	2 mm	4 h, RT	<LOD*^b^*
Dimethyl trisulfide	100 μm	4 h, RT	<LOD
Lipoic acid	100 μm	4 h, RT	<LOD
Dihydrolipoic acid	100 μm	4 h, RT	<LOD
Na_2_S_2_O_3_	100 μm	4 h, RT	<LOD
Na_2_S_2_O_4_	100 μm	4 h, RT	<LOD
NADH	5 mm	4 h, RT	<LOD
NADPH	5 mm	4 h, RT	<LOD
NaSCN	100 μm	4 h, RT	<LOD
NaNO_2_	100 μm	4 h, RT	<LOD
NO	50 μm	4 h, RT	<LOD
ONOO^−^	100 μm	4 h, RT	1.25
H_2_O_2_	100 μm	4 h, RT	<LOD
*t*-BuOOH	100 μm	4 h, RT	<LOD
O_2_^˙̄^	1.79 nmol of cytochrome *c*/min	4 h, RT	<LOD
HOCl	100 μm	4 h, RT	<LOD

*^a^* RT, room temperature.

*^b^* LOD, limit of detection.

**Figure 3.**
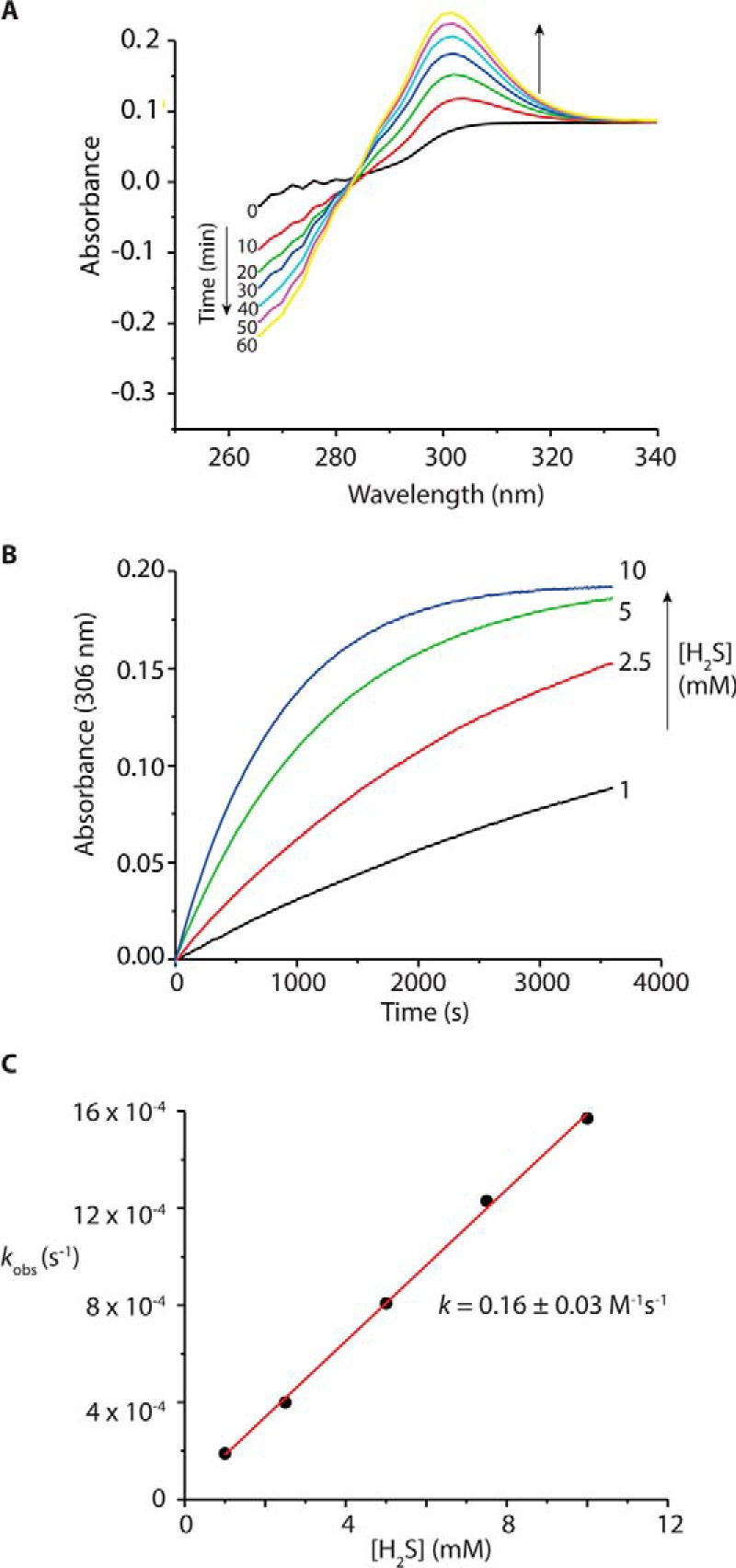
**Rate of reaction of MitoA with H_2_S to form MitoN.** *A,* differential time-resolved spectra where 40 μm MitoA was mixed with 2.5 mm H_2_S, with MitoA serving as the blank. The spectra illustrate the time-dependent disappearance of the MitoA peak at ∼270 nm and the appearance of a new peak assigned to MitoN at ∼306 nm. *B,* kinetic traces at 306 nm obtained for the reaction of 40 μm MitoA with increasing concentrations of H_2_S, the absorbance was observed over 6 min at 306 nm using a Hewlett Packard 8452A Diode Array Spectrophotometer under pseudo-first order conditions. *C,* plot of *k*_obs_ *versus* [H_2_S]. The experiments were performed in 300 mm potassium phosphate buffer, pH 7.4, at 22 °C in triplicates.

We next measured the rate of reaction of MitoA with H_2_S from the time-dependent disappearance of the MitoA peak at ∼270 nm and the appearance of a new peak at 306 nm due to MitoN (Fig. 3*A*). The absorption maxima for the non-TPP aromatic rings were identified using 4-azido-*N*-(hex-1-yl)benzamide and 4-amino-*N*-(hex-1-yl)benzamide, which are analogues of MitoA and MitoN, respectively, but without the TPP targeting group. We then incubated MitoA with known concentrations of H_2_S and measured the formation of MitoN at 306 nm (Fig. 3*B*). From these we were able to plot the observed initial rate constant (*k*_obs_) for MitoN formation against the concentration of H_2_S and calculate a rate constant: 0.16 ± 0.03 m^−1^ s^−1^ at 22 °C (Fig. 3*C*). This compares with literature rates for the reaction of H_2_S with phenyl azides: 0.12 m^−1^ s^−1^ at 10 °C and 0.95 m^−1^ s^−1^ at 25 °C (37). One concern about the use of MitoA is that its reaction consumes H_2_S and generates H_2_S_2_ (37), thereby potentially disrupting H_2_S signaling pathways and altering protein persulfidation. Hence the slow reaction of MitoA with H_2_S is advantageous because it allows selective detection, while also minimizing disruption to endogenous H_2_S signaling pathways. Together these data indicate that, in agreement with other aryl azides (45, 46), MitoA reacts selectively with H_2_S and therefore that the conversion of MitoA to MitoN can be used to assess H_2_S *in vivo*.

### Analysis of MitoA and MitoN by LC-MS/MS

**Figure 4.**
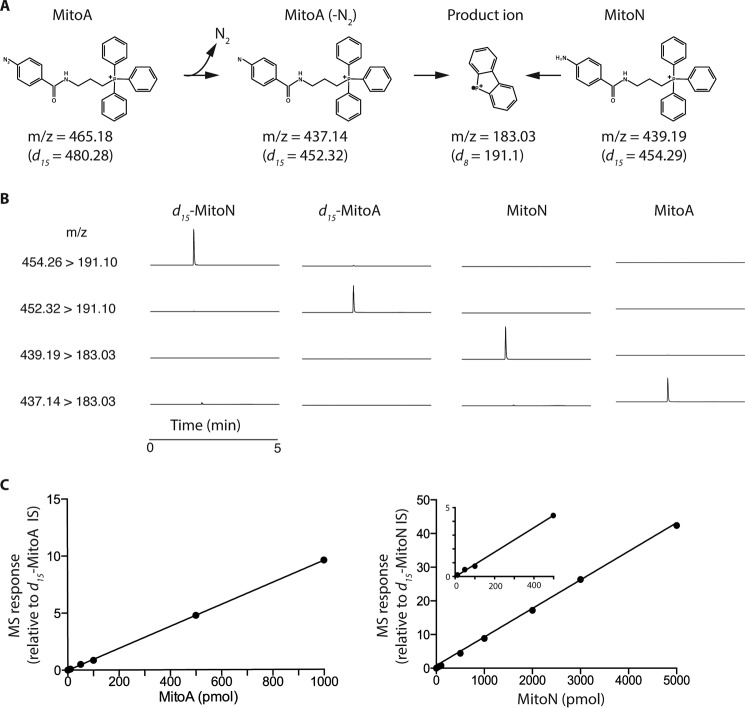
**Detection of MitoA and MitoN by LC-MS/MS.** *A,* the transitions used for analysis of MitoA and MitoN, and their deuterated derivatives, are shown. *B,* typical chromatograms showing the *m*/*z* transitions measured simultaneously for 50 pmol of *d*_15_-MitoA, MitoA, *d*_15_-MitoN, or MitoN. Each trace is normalized to the highest total ion count peak within that trace. *C,* typical standard curves for MitoA and MitoN detection by LC-MS/MS. Samples were prepared in 20% ACN, 0.1% FA and contained known MitoA or MitoN concentrations and their deuterated internal standards. Each point is mean ± range of duplicate measurements.

To be useful as an *in vivo* mass spectrometric probe MitoA and MitoN have to be extracted from tissues and analyzed by LC-MS/MS, relative to deuterated internal standards (IS) (47–49). To establish the LC-MS/MS assay we first determined fragmentation conditions that enabled sensitive detection of MitoA and MitoN, and their deuterated internal standards (Fig. 4*A*). For the analysis of MitoA we took into account the neutral loss of N_2_ from MitoA to generate the nitrene, which under these conditions was complete upon entrance to the first mass spectrometer quadrupole. Using the transitions shown in Fig. 4*A* we could detect MitoA and MitoN, and their deuterated IS, selectively by LC-MS/MS (Fig. 4*B*). Hence we were able to generate standard curves for MitoA and MitoN down to picomole levels (Fig. 4*C*).

A potential concern for the LC-MS/MS analysis is that MitoA might react with H_2_S generated upon homogenization and extraction of tissues, for example, by release of labile sulfur from iron-sulfur centers. The possibility of H_2_S release was minimized by omitting acid during tissue extraction. Even so, to assess whether an artifactual reaction with released H_2_S could occur, we incubated MitoA in tissue extraction medium supplemented with H_2_S for up to 4 h, and then measured MitoN formation by LC-MS/MS. As expected, this showed some conversion of MitoA to MitoN, but with a very small (∼0.02) increase in the MitoN/MitoA ratio (supplemental Fig. S4). Considering the high H_2_S concentration added, and that the incubation time of 4 h was far longer than that used during conventional extraction, we conclude that the artifactual conversion of MitoA to MitoN during tissue extraction is negligible. In any case such a potential artifact would be equal for all tissue samples and therefore would not affect comparisons. To conclude, we have established a sensitive LC-MS/MS assay for H_2_S that can be applied to tissues.

### Uptake of MitoA and MitoN by mitochondria and cells

**Figure 5.**
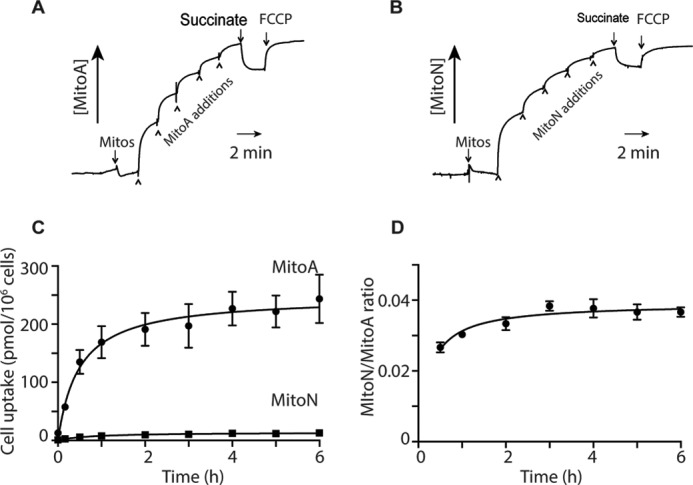
**Accumulation of MitoA and MitoN by isolated mitochondria and cells.** The uptake of MitoA (*A*) and MitoN (*B*) was examined in isolated mitochondria (2 mg of protein/ml) incubated in KCl medium supplemented with 4 μg/ml of rotenone and 100 nm nigericin using a TPP-selective electrode. The compounds were added in 1 μm steps, followed by 10 mm succinate and 500 nm FCCP. *C,* MitoA uptake by HCT116 cells. HCT116 cells were plated at 250,000 cells/well (∼27,000 cells/cm^2^) in 6-well plates overnight and then incubated with 10 μm MitoA in DMEM containing 10% FBS and antibiotics. At various times 1 ml of supernatant was removed for analysis, the rest was discarded, and the cells were rinsed with 1 ml of PBS and collected by scraping into 0.5 ml of PBS and pelleted by centrifugation (16,000 × *g*, 3 min, room temperature). Cell pellets were snap frozen and stored at −20 °C. Compounds were extracted and quantified by LC-MS/MS. Results are mean ± S.E. for *n* = 3. MitoA levels in the supernatant did not change over 6 h (data not shown). *D,* for the experiment in *C*, the level of MitoN in the cell pellets were assessed in parallel with MitoA and the MitoN/MitoA ratio is shown.

To be useful for the analysis of H_2_S *in vivo*, MitoA must be taken up into mitochondria in response to the membrane potential. To see if this occurred we next assessed the accumulation of MitoA and MitoN by isolated mitochondria using a triphenylphosphonium (TPP)-selective electrode (54) (Fig. 5, *A* and *B*). Both probes accumulated inside mitochondria ∼650- to ∼1,000-fold in response to the membrane potential and this uptake was reversed by abolishing the mitochondrial membrane potential with the uncoupler FCCP. Therefore, MitoN and MitoA are taken up by energized mitochondria as expected for TPP compounds. To determine whether MitoA was accumulated within cells we first established that the toxicity of MitoA or MitoN to cells *in vitro* was negligible at concentrations up to 12.5 μm, determined by following the effects of the compounds on cell proliferation (supplemental Fig. S5). To assess the uptake of MitoA into cells, we incubated HCT116 cells with MitoA and measured the amounts taken up by the cells over time by LC-MS/MS (Fig. 5*C*). This showed that MitoA was taken up into cells over time, coming to a steady state after ∼1 h. Over this time the amount of MitoN generated was small (Fig. 5*C*), consequently the MitoN/MitoA ratio stayed low and constant throughout the incubation (Fig. 5*D*). Therefore MitoA is taken up by cells and, at least under these conditions, there was low MitoN formation.

### Reactivity and stability of MitoA in tissue homogenates

**Figure 6.**
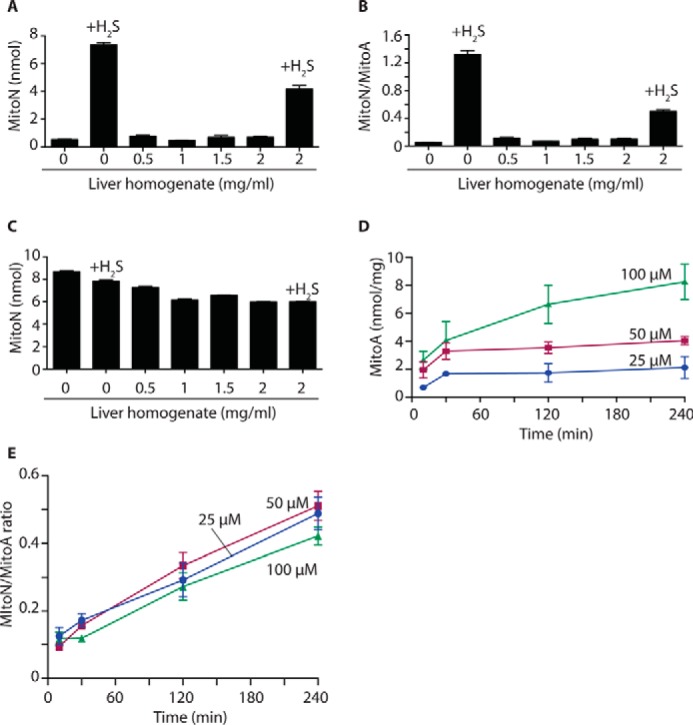
**MitoA and MitoN metabolism in the liver *ex vivo*.** *A* and *B,* MitoA metabolism by a liver homogenate. MitoA (10 μm) was incubated with various amounts of liver homogenate, or with liver homogenate and NaHS (25 μm), for 2 h at 37 °C. Samples were then analyzed by LC-MS/MS to quantify MitoA (data not shown), MitoN (*A*), and the MitoN/MitoA ratio (*B*). Data are mean ± S.E., *n* = 3. *C,* MitoN metabolism by a liver homogenate. MitoN (10 μm) was incubated with various amounts of liver homogenate, or with liver homogenate and NaHS (25 μm), for 2 h at 37 °C. Samples were then analyzed by LC-MS/MS for MitoN. Data are mean ± S.E., *n* = 3. *D,* uptake of MitoA by liver sections. Rat liver sections of ∼20 to 50 mg wet weight were incubated with MitoA in University of Wisconsin solution at 4 °C. After incubation, the tissue pieces were rinsed in PBS, dried, and snap frozen prior to quantification of MitoA and MitoN by LC-MS/MS. Data show the accumulation of MitoA over time and are mean ± S.E., *n* = 3. *E,* MitoN/MitoA ratio in liver sections. The MitoN/MitoA ratio was determined over time in liver sections as described in *D*.

MitoA is designed to assess H_2_S in tissues *in vivo*. To determine whether there were any enzymatic processes in tissues that could convert MitoA to MitoN in the absence of H_2_S, we incubated MitoA with liver homogenates and measured MitoN formation (Fig. 6*A*). Under these conditions enzymes are available to react with MitoA, but the lack of tissue and cell architecture means that any H_2_S generated will diffuse away. Incubation of MitoA with a liver homogenate led to a negligible MitoN formation (Fig. 6*A*) and no increase in the MitoN/MitoA ratio (Fig. 6*B*), even though the reaction proceeded readily when H_2_S was added (Fig. 6, *A* and *B*). Similarly, incubation of MitoN with a liver homogenate in the presence or absence of H_2_S led to very little loss of the compound (Fig. 6*C*). Together these data suggest that MitoA conversion to MitoN in tissues is negligible in the absence of H_2_S, and that once formed MitoN is stable.

### Response of MitoA to H_2_S in ischemic liver sections

The next step was to see how MitoA responded to an increase in endogenous H_2_S within a tissue. To assess this we incubated liver tissue sections with various concentrations of MitoA at 4 °C, designed to mimic conditions during organ storage for transplantation (55, 56). MitoA was taken up into the tissue sections over time (Fig. 6*D*). Under these conditions the tissue will be largely ischemic, as there is no perfusion and O_2_ will be depleted by the oxidation of endogenous substrates. The lack of O_2_ will block the activity of the mitochondrial SQR, which is the major way in which H_2_S is degraded *in vivo* (Fig. 1*A*). It is known that when the activity of the SQR is decreased by depletion of the mitochondrial CoQ pool, that there is an accumulation of H_2_S within mice *in vivo* (57). Thus, under ischemic conditions the inhibition of SQR is expected to increase H_2_S levels. Furthermore, H_2_S production by the mitochondrial 3-mercaptopyruvate sulfurtransferase enzyme is driven by the reduced form of thioredoxin, which will also increase in concentration upon ischemia (58). Therefore it is likely that there will be an increase in H_2_S levels in these liver sections upon ischemia.

To see if this increase in H_2_S led to MitoN formation in the ischemic liver sections, we measured MitoN levels over time and expressed these relative to MitoA. This showed an increase in the MitoN/MitoA ratio over time (Fig. 6*E*), consistent with increased H_2_S formation in ischemic organs over time. Importantly, the MitoN/MitoA ratios at a given time point were similar for all initial MitoA concentrations used. Thus the ratiometric analysis of MitoN relative to MitoA corrects for differences in MitoA content and suggests that the MitoA/MitoN ratio is a robust measure of changes in H_2_S levels.

### Uptake and metabolism of MitoA and MitoN within tissues in vivo

**Figure 7.**
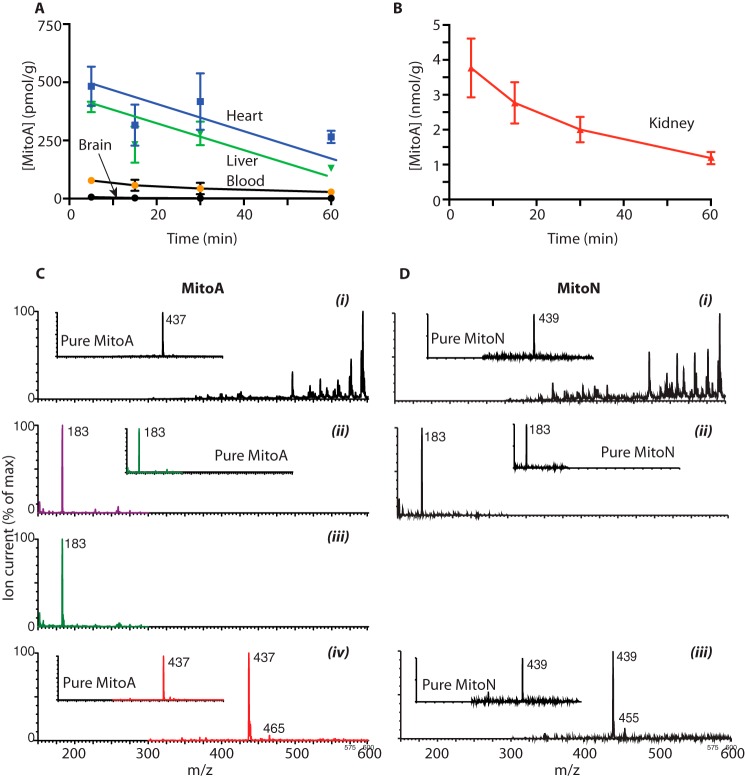
**Uptake and metabolism of MitoA *in vivo*.** *A* and *B,* mice were injected with 50 nmol of MitoA and tissues were collected, extracted, and analyzed by LC-MS/MS. Results are mean ± S.E., *n* = 3. *C*, MitoA (50 nmol) was administered to mice. After 60 min the liver was collected, extracted, and prepared as for mass spectrometry (LC-MS/MS) but omitting the internal standards and analyzed by mass spectrometry by direct infusion at 5 μl/min. *C*, *i,* shows the mass spectrum of the liver extract. *ii,* shows the MS/MS spectrum of the liver extract assessed for MitoA (437 > 183). The *inset* shows the MS/MS spectrum for pure MitoA. *iii,* shows the MS/MS spectrum of the liver extract assessed for MitoN (439 > 183). The *inset* shows the MS/MS spectrum for pure MitoN. *iv,* shows the precursor scan of the liver extract assessed for precursor ions that generate a product ion of *m*/*z* 183. The *inset* shows the precursor ion scan for pure MitoA. *D,* MitoN (50 nmol) was administered to mice. After 60 min the liver was collected, extracted into solvent, and prepared as for mass spectrometry, but omitting the internal standards, and analyzed by MS by direct infusion at 5 μl/min. *D*, *i*, shows the mass spectrum of the liver extract. *ii*, shows the MS/MS spectrum of the liver extract assessed for MitoN (439 > 183). The *inset* shows the MS/MS spectrum for pure MitoN. *iii*, shows the precursor scan of the liver extract assessed for precursor ions that generate a product ion of *m*/*z* 183. The *inset* shows the precursor ion scan for pure MitoN. For all spectra the maximum ion counts are indicated on the spectra and all ion counts are expressed as % of the maximum.

Previously developed exomarkers such as MitoB were administered to mice as a single intravenous (i.v.) bolus injection (47). This approach enabled the compounds to be very rapidly accumulated within tissues *in vivo*, after which they are gradually lost over the following hours. This protocol gives sufficient time for the accumulated compound (*e.g.* MitoB) to react with its target (*e.g.* H_2_O_2_) to form an exomarker (*e.g.* MitoP). To see if MitoA could also be used in this way, we assessed MitoA distribution within mouse tissues following a single i.v. injection (Fig. 7, *A* and *B*). This showed rapid clearance of MitoA from the blood and its accumulation into the heart and liver, but not the brain, as expected for TPP compounds in mice (59, 60) (Fig. 7*A*). The kidney accumulated more MitoA than the other tissues, presumably due to direct removal of the initial bolus from the circulation, as well as redistribution of MitoA from other tissues over time (Fig. 7*B*). MitoA was gradually lost from the tissues over time (Fig. 7, *A* and *B*), consistent with other TPP compounds (59). These findings suggest that MitoA is accumulated rapidly by tissues and is retained long enough to respond to local levels of H_2_S.

To report on H_2_S levels within tissues *in vivo* MitoA should only be converted to MitoN by reaction with H_2_S, with minimal side reactions. To assess this we injected MitoA into mice and 1 h later isolated the liver. The liver was chosen because it readily accumulates MitoA (Fig. 7*A*) and has extensive xenobiotic metabolism. Thus, if MitoA is biotransformed to compounds other than MitoN *in vivo* the liver should demonstrate this biotransformation. Extraction and MS analysis of the liver showed a large number of compounds (Fig. 7*C, i*). Analysis by MS/MS at the transition diagnostic for MitoA (437 > 183) showed the presence of MitoA (Fig. 7*C, ii*). Similarly, analysis at the transition diagnostic for MitoN (439 > 183) showed some MitoN formation *in vivo* (Fig. 7*C, iii*). These mass spectra were identical to those of pure MitoA and MitoN (Fig. 7*C, ii* and *iii*, *insets*). To see if MitoA and MitoN were metabolized in the liver to other TPP containing molecules, we next carried out a precursor scan of the extract to identify all compounds that fragmented to a product with *m*/*z* 183, diagnostic of a TPP-containing compound (47, 61). In the liver extract the only precursor molecules that gave a product ion of *m*/*z* 183 corresponded to the nitrene product of MitoA upon neutral loss of N_2_ (*m*/*z* 437), MitoN (*m*/*z* 439), with a small amount of intact MitoA (*m*/*z* 465) (Fig. 7*C, iv*). This precursor ion pattern was similar to that of pure MitoA (Fig. 7*C, iv*, *inset*).

Another potential concern is whether MitoN could be further metabolized *in vivo*, following its formation from MitoA. To assess this we injected MitoN into mice and 1 h later isolated the liver and analyzed this extract by MS analysis (Fig. 7*D, i*). Analysis by MS/MS at the transition diagnostic for MitoN (439 > 183) showed the presence of MitoN (Fig. 7*D, ii*), and the spectrum was identical to that of pure MitoN (Fig. 7*D, ii, inset*). To see if MitoN was metabolized within the liver *in vivo*, we next carried out a precursor scan of the extract to identify all compounds that fragmented to a product with *m*/*z* 183, diagnostic of a TPP containing compound (47, 61). In the liver homogenate the only parent molecules that gave a daughter ion of *m*/*z* 183 corresponded to MitoN (*m*/*z* 439), with a small peak at 455 (Fig. 7*D, iii*). This precursor ion pattern was similar to that of pure MitoN (Fig. 7*D, iii*, *inset*). The precursor ion at 455 is a +16 shift from MitoN and may represent oxidation of MitoN by addition of an oxygen atom. Even so, it suggests that once formed MitoN is relatively stable and that any further metabolism of MitoN is unlikely to affect the analysis of H_2_S *in vivo*.

### Use of MitoA to analyze H_2_S in the ischemic heart in vivo

**Figure 8.**
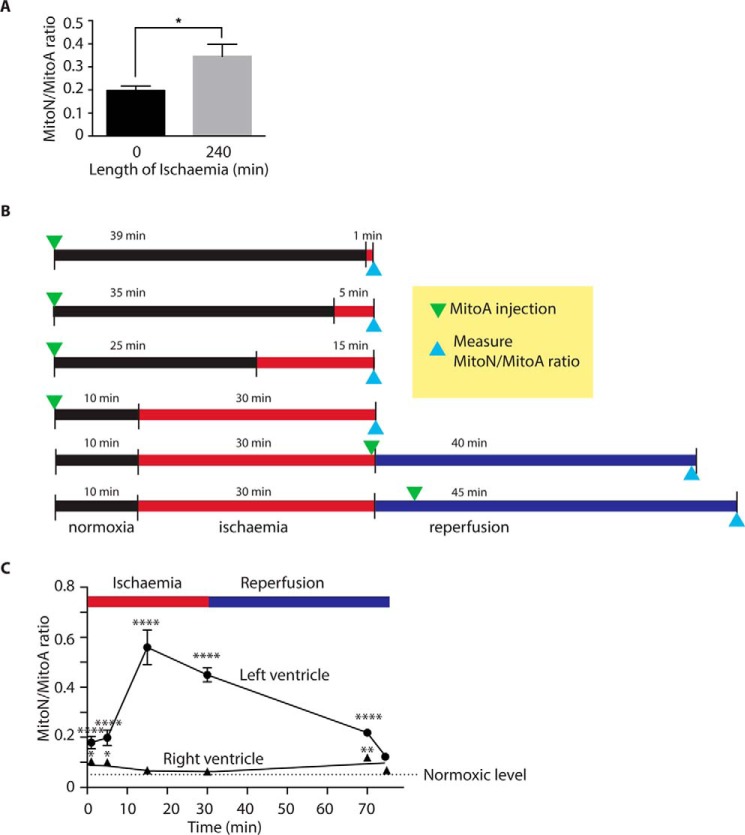
**Assessment of H_2_S *in vivo* using MitoA.** *A,* MitoA in the heart during storage *ex vivo*. MitoA (50 nmol) was administered to mice via tail vein injection. The heart was removed after ∼12 min of warm ischemia. The hearts were then either snap frozen or stored for 240 min on ice and then snap frozen. Samples were analyzed by LC-MS/MS for MitoA and MitoN, and the MitoN/MitoA ratio was calculated. Results are mean ± S.E., *n* = 3. *, *p* < 0.05 by Student's two-tailed *t* test. *B,* protocol for the LAD artery occlusion experiments. *C,* mice were injected with 50 nmol of MitoA prior to ischemia of the left ventricle induced by occlusion of the LAD artery. At the end of the protocol the hearts were separated into right (normoxic) and left (normoxic or ischemic) ventricles, snap frozen, and analyzed for MitoA and MitoN content by LC-MS/MS, enabling the MitoN/MitoA ratio to be calculated. Data are mean ± S.E., *n* = 3. *, *p* < 0.05; **, *p* < 0.01; ***, *p* < 0.001; ****, *p* < 0.0001, by two-way analysis of variance followed by post hoc Bonferroni test.

The increase in the MitoN/MitoA ratio in the ischemic liver (Fig. 6*E*) is consistent with an increase in H_2_S in tissue during ischemia. To extend this, we next focused on the heart as its metabolism changes dramatically upon ischemia (62, 63). As a first step to see whether there was any accumulation of H_2_S in the ischemic heart we administered MitoA to mice, isolated the heart, and stored it under ischemic conditions mimicking those used for organ storage during transplantation (Fig. 8*A*). The MitoN/MitoA ratio increased after 240 min ischemia, consistent with elevated H_2_S during the ischemia inherent in organ storage.

To explore further how H_2_S levels respond to ischemia we next used the left anterior descending (LAD) model of acute myocardial infarction. MitoA was injected into mice and then cardiac ischemia was induced by closing off the LAD artery, thus subjecting a large section of the left ventricle to ischemia. In previous work we have shown that when the occlusion to the LAD artery was removed after 30 min ischemia and the tissue reperfused with oxygenated blood, there was extensive ischemia-reperfusion injury (63). This model thereby enables us to use MitoA to assess variations of H_2_S levels during both ischemia and reperfusion. In comparing the formation of MitoN through these changes it is important that MitoA is present in the tissue for the same duration. To ensure this, we injected MitoA at various time points before or after initiation of ischemia (Fig. 8*B*). Importantly, this protocol meant that MitoA was exposed to a range of times of ischemia and reperfusion, whereas always being present in the tissue for 40 min, thereby facilitating comparison. In the LAD model only the left ventricle is exposed to ischemia and reperfusion, however, as it was unclear what effect ischemia in the left ventricle would have on the right ventricle from the same mouse, the right ventricle from the same mouse cannot act as a control. Therefore the left and right ventricles from mice that underwent ischemia were compared with the appropriate ventricle from MitoA-treated normoxic mice.

When MitoA was injected into control mice in which the hearts were not exposed to ischemia or reperfusion, analysis of the right and left ventricles 40 min later showed a low MitoN/MitoA ratio in both ventricles (Fig. 8*C*). When MitoA was injected before reperfusion, the MitoN/MitoA ratio was increased compared with normoxic hearts, presumably as result of H_2_S present in the tissue before reperfusion. However, administration 5 min after reperfusion resulted in the MitoN/MitoA ratio similar to normoxic hearts. Furthermore, during ischemia there was a dramatic increase in the MitoN/MitoA ratio in the left ventricle, but not in the right (Fig. 8*C*). The MitoN/MitoA ratio increased the longer the duration of ischemia to which the left ventricle was exposed (Fig. 8*C*). Comparison between the ischemic and non-ischemic zones of the heart showed that the H_2_S increase was local to the ischemic tissue area, and did not diffuse to surrounding tissue of the same organ. Together, these findings are consistent with an increase in H_2_S during ischemia that is rapidly reversed once oxygen is reintroduced into the tissue upon reperfusion. Furthermore, these findings indicate that MitoA can be used to assess changes in H_2_S *in vivo*.

## Conclusions

Here we have applied the exomarker approach to the analysis of H_2_S *in vivo*. To do this we used the well established chemistry of aryl azides and their conversion to an amine on reaction with H_2_S (33, 36, 46). The combination of this chemistry with mitochondria targeting by the TPP moiety led to its rapid uptake into cells *in vivo* where it is predicted to be mainly located in the mitochondria. There, MitoA should react with H_2_S to form the diagnostic exomarker MitoN. The chemical selectivity for H_2_S of the MitoA probe was clear, and it did not react with other potential factors found in biological environments. Most importantly, it did not react directly with persulfides, suggesting that *in vivo* it reports on free H_2_S independently of any changes in the levels of protein or low molecular weight persulfides.

The most important aspect of the development of MitoA is that it enables the assessment of changes in H_2_S *in vivo*. This exomarker approach has previously been used to infer levels of evanescent species such as H_2_O_2_ in a range of living organisms, where assessments *ex vivo* or in isolated cells would have introduced confounding factors making the data hard to interpret. The experimental constraints are similar with assessment of free H_2_S in tissues, and are in fact exacerbated due to the rapid diffusion of H_2_S from *in vitro* systems, such as cell culture where H_2_S rapidly disperses into the large amount of medium above a cell monolayer and is then lost into the head space. In contrast, in tissues the situation is quite different, where the local architecture can enable a large build up of H_2_S. Consequently, measurements of H_2_S levels within tissues *in vivo* under normal and pathological conditions are essential to infer the roles of H_2_S *in vivo*.

As a proof of principle to test MitoA *in vivo*, we assessed changes in H_2_S in tissues undergoing ischemia/hypoxia. Ischemia is expected to lead to an increase in H_2_S due to the inhibition of H_2_S degradation by SQR that occurs in the absence of O_2_. An advantage of this model to test the efficacy of MitoA was that there was no intervention with an exogenous agent or H_2_S donor, thus it indicates that MitoA can respond to H_2_S levels that may arise *in vivo* under pathological conditions. These findings also demonstrated that there were dramatic changes in H_2_S between the ischemic and normoxic regions of the heart, and that the H_2_S levels rapidly returned to normal upon reperfusion with oxygenated blood. None of these assessments would have been possible prior to the development of MitoA. Interestingly, as well as enabling the testing of MitoA, these findings suggested that H_2_S levels may be a significant aspect of how tissues respond to ischemia and these changes in H_2_S will be explored further as a potential ischemic signaling pathway.

There are a few limitations to the exomarker approach. Because the ratio of MitoN to MitoA can only be determined from tissue samples *ex vivo*, each time point requires killing a mouse. Furthermore, the analysis is inevitably an average of many different cells and mitochondria. Consequently, complementary techniques are required to assess changes in individual cells and in real time. Another caveat is that many of the effects of altering H_2_S metabolism are likely to be caused by changes in protein persulfidation that will not be picked up directly by the MitoA approach, which only responds to free H_2_S. Therefore it is important in the future to develop related techniques to assess protein persulfidation in parallel to the measurement of free H_2_S. Finally, it is difficult to relate the MitoN/MitoA ratio to the actual amount of H_2_S present *in vivo*.

In summary, we have developed a mass spectrometric probe that will enable for the first time changes in the levels of H_2_S to be assessed *in vivo*. Use of this probe will greatly facilitate investigations of the role of H_2_S in health and disease.

## Experimental procedures

### Chemical syntheses

MitoA and MitoN were synthesized from triphenylphosphine and the deuterated analogues, *d*_15_-MitoA and *d*_15_-MitoN, from tri(pentadeuterophenyl)phosphine as shown in supplemental Fig. S1A. The triarylphosphines were each reacted with 1,3-diodopropane to give iodopropyl-TPP salts **1** and ***d*_15_-1**. Displacement of the alkyl iodide by azide gave the azidopropyl-TPP salts **2** and ***d*_15_-2**, and hydrogenation converted these into the amines **3** and ***d*_15_-3**. The latter were coupled with 4-azidobenzoic acid using diisopropylcarbodiimide in acetonitrile (ACN) and the resulting amide precipitated from solution in each case. Ion exchange then gave MitoA and *d*_15_-MitoA as their mesylate salts in moderate yield. In a similar way, coupling 4-aminobenzoic acid followed by ion exchange gave MitoN and *d*_15_-MitoN as their mesylate salts. 4-Azido-*N*-(hex-1-yl)benzamide **4** and 4-amino-*N*-(hex-1-yl)benzamide **5**, which are analogues of MitoA and MitoN lacking the TPP targeting group, were prepared for comparison (supplemental Fig. S1B). Further details of the chemical syntheses are given under supplementary materials. Peroxynitrite was prepared according to Refs. 64 and 65. A persulfide analogue of *S*-nitrosopenicillamine, NAP-SSH, was synthesized as described (52) and was prepared as a 1 mm stock in 10 mm HCl at 4 °C.

### Assessment of compound properties

MitoA and MitoN stock solutions (5 mm in ethanol (EtOH)) were stable for at least 6 months at −20 °C. Absorbance spectra were carried out in 1-ml cuvettes in KCl buffer (120 mm KCl, 10 mm Hepes and 1 mm EGTA, pH 7.2 (KOH)) using a Shimadzu UV-2501PC UV-visible spectrophotometer. The molar extinction coefficient for MitoA at 267 nm in KCl buffer was calculated gravimetrically: 20.4 ± 0.1 × 10^3^ m^−1^ cm^−1^.

Reversed phase-high performance liquid chromatography (RP-HPLC) was done using a Gilson 321 pump with attached UV-visible 151 system (Gilson). Samples were filtered through a 0.22-μm polyvinylidene difluoride (PVDF) filter (Millex, Millipore), loaded, and run over a C_18_ column (Jupiter 300 Å, Phenomenex) with a Widepore C_18_ guard column (Phenomenex). HPLC buffer A (0.1% (v/v) trifluoroacetic acid (TFA) in water) and HPLC buffer B (0.1% (v/v) TFA/acetonitrile) were used with the gradient: 0–2 min, 5% B; 2–17 min, 5–100% B; 17–19 min, 100% B; 19–22 min 100–5% B.

### Cell culture

Cells (C2C12 or HCT116 from the American Type Culture Collection) were incubated at 37 °C in a humidified atmosphere of 95% air and 5% CO_2_ in Dulbecco's modified Eagle's medium (Invitrogen) supplemented with 10% (v/v) fetal bovine serum (FBS) and 100 units/ml of penicillin and 100 μg/ml of streptomycin.

### Preparation of H_2_S solutions

Unless indicated otherwise, standard solutions of H_2_S were prepared by dissolving NaHS in KCl buffer, the pH was re-adjusted to 7.2 and then the solution was aliquoted into tubes with no head space and kept on ice. The H_2_S concentration was determined by the methylene blue assay (66).

### Mitochondrial preparation and incubation

Liver mitochondria were prepared from female Wistar rats killed by stunning and cervical dislocation, followed by homogenization of the liver and differential centrifugation in ice-cold STE buffer (250 mm sucrose, 10 mm Tris, 1 mm EGTA, pH 7.4). The protein concentration was determined by the biuret assay using bovine serum albumin as a standard (67).

### Extraction of MitoA and MitoN

For cells, frozen pellets were thawed, vortexed, and resuspended in 210 μl of 95% acetonitrile (ACN) spiked with internal standard (100 pmol of *d*_15_*-*MitoA and 100 pmol of *d*_15_*-*MitoN). Following protein precipitation (30 min, 4 °C) and centrifugation (16,000 × *g*, 10 min, room temperature) the supernatant was filtered through a 0.22-μm filter and dried under vacuum (miVac Quattro concentrator; Genevac) and stored at −20 °C. For extraction from tissues, ∼50 mg wet weight tissue was placed in a 2-ml Eppendorf tube containing 210 μl of 95% ACN spiked with internal standards (100 pmol of *d*_15_*-*MitoA and 100 pmol of *d*_15_*-*MitoN). For the blood no further additions were made, but for the tissues we added ∼50 μl of beads (liver, brain, and kidney, 0.5-mm diameter zirconium oxide beads; heart, 0.9–2.0-mm diameter stainless steel beads, both from Next Advance) and homogenized for 3 min using a Bullet Blender (Storm 24; Next Advance) at speed 8. Samples were centrifuged (16,000 × *g*, 10 min, room temperature), the supernatant was transferred to a new Eppendorf tube. The pellet was re-extracted with 95% ACN, and the supernatants were pooled and incubated for 30 min at 4 °C to precipitate proteins. After centrifugation (16,000 × *g*, 10 min, room temperature) the supernatant was filtered and dried as above and stored at −20 °C. Prior to analysis the samples were resuspended in 20% ACN, 0.1% FA, centrifuged (16,000 × *g*, 10 min, room temperature), and transferred to mass spectrometry vials (TrueView^TM^ LCMS Certified, Waters).

### LC-MS/MS analysis

For MS/MS analysis we used a triple-quadrupole mass spectrometer (Waters Xevo TQ-S under positive ion mode: source spray voltage, 3.2 kV; cone voltage, 125 V; ion source temperature, 100 °C; collision energy, 75 V). Nitrogen and argon were used as curtain and collision gas, respectively. MS fragmentation patterns were determined by direct infusion (5 nm at 50 μl/min in 20% ACN, 0.1% FA). For LC-MS/MS analyses the mass spectrometer was connected in series to an I-class Acquity LC system (Waters). Samples were stored in an autosampler at 4 °C and 2-μl samples went into a 15-μl flow-through needle and RP-UPLC at 40 °C using an Acquity UPLC® BEH C18 column (1 × 50 mm, 1.7 μm; Waters) with a Waters UPLC filter (0.2 μm). MS buffers A (95% water, 5% ACN, 0.1% FA) and B (90% ACN, 10% water, 0.1% FA) were infused at 200 μl/min using the following gradient: 0–0.3 min, 5% B; 0.3–3 min, 5–100% B; 3–4 min, 100% B, 4.0–4.10, 100–105% B; 4.10–4.60 min, 5% B. Eluant was diverted to waste from 0 to 1 min and from 4 to 4.6 min. Compounds were detected in multiple reactions monitoring in positive ion mode. Under these conditions MitoA underwent neutral loss of N_2_ to a nitrene, which was used as the precursor ion. For quantification the following transitions were used: MitoA, 437 > 183; *d*_15_-MitoA, 452 > 191; MitoN, 439 > 183; *d*_15_-MitoN, 454 > 191. Standard curves with known amounts of MitoA and MitoN were prepared, spiked with IS, and extracted following the protocol outlined above. The peak area of MitoA, MitoN, and IS of samples and standard curves were quantified using the MassLynx 4.1 software.

### Liver homogenate incubations

Liver was collected from a female Wistar rat that had been killed by stunning and cervical dislocation, and carried out in accordance with the UK Home Office Guide on Animal Experiments. The liver was transferred to ice-cold STE buffer, cut into small pieces using a razor blade, washed in ice-cold KCl buffer, and homogenized in a Dounce homgenizer, and the protein concentration was determined by biuret assay.

### Mouse experiments

Wild-type male C57BL/6J mice (8–10 weeks old) were obtained from Charles Rivers Laboratories (UK). Mice were housed under standard laboratory conditions with food and water available *ad libitum* and experiments were carried out in accordance with the UK Home Office Guide on Animal Experiments and were approved under Project Licenses PPL 70/8238, 70/7963, and PPL 80/2638. To assess MitoA uptake and distribution *in vivo*, mice were placed in a heating chamber set to 38 °C (Vet-Tech Solutions) for ∼5 min, inserted in a rodent restrainer with free access to the tail, and MitoA (50 nmol) was injected into a lateral tail vein in 100 μl of saline through a 27-gauge needle connected to a 1-ml syringe. Mice were then monitored throughout the incubation period until time points for tissue collection were reached.

For the *ex vivo* incubation of hearts, mice were anesthetized with isoflurane (Abbott Laboratories, US) and oxygen at 2 liters/min. MitoA (50 nmol) was administered in saline via the inferior vena cava and then the heart was explanted as described (68). In brief, 100 μl of heparin was administered prior to exsanguination by division of the inferior vena cava and aorta. The inferior vena cava, hemiazygous vein, superior vena cava, innominate, left carotid, and left brachiocephalic were ligated prior to division of the descending aorta, pulmonary artery, and pulmonary veins. The heart was perfused with 500 μl of ice-cold Soltran (Baxter Healthcare, UK). During dissection and ligation of vessels the heart was topically cooled with 4 °C saline every 2 min. This process took 12 min and the heart was excised and stored in 10 ml of Belzer UW® Cold Storage Solution, University of Wisconsin solution (Belzer UW® Cold Storage Solution, Bridge to Life Ltd.) on ice for 240 min or snap frozen immediately. Samples were stored at −80 °C until extraction for LC-MS/MS.

*In vivo* IRI was performed by using an open-chest, *in situ* mouse (C57BL/6J) heart model in which the LAD is surgically transiently occluded as recently described (69). Mice were anesthetized (pentobarbital sodium; 70 mg/kg; intraperitoneal (i.p.)), intubated endotracheally, and ventilated with 3 cm of H_2_O positive end-expiratory pressure (110 breaths per min, 125–150 ml tidal volume) using a mouse ventilator (MINIVENT Type 845, Harvard Apparatus, Germany). Body temperature was monitored by rectal probe and maintained at 37 °C via an animal temperature controller (TCAT-2LV, Physitemp, Clifton, NJ). Corneal and withdraw reflexes were checked for anesthesia depth and additional anesthesia was administered throughout the experimental protocol if needed. A left thoracotomy was performed and the heart was exposed via stripping of the pericardium. Regional ischemia was then induced via ligation of the main branch of the LAD. Successful occlusion was confirmed by color change of the anterior wall of the left ventricle and apex from a bright red color typical of a perfused heart to off-white and ischemia was sustained for 30 min, at which point the tissue was reperfused. Reperfusion of the heart was confirmed by a color change of the ischemic zone to red. Control mice were sham treated. MitoA (50 nmol) was administered as a 100-μl bolus via the tail vein at various times during the procedure. Tissue was collected, snap frozen, and stored at −80 °C until analysis.

## Author contributions

S. A. characterized MitoA and MitoN. C. B.-G. synthesized MitoA and MitoN. A. L. and S. A. carried out the mass spectrometric analyses. T. R. carried out the mouse LAD experiments. T. A. P. processed samples for mass spectrometry and carried out the ion-selective electrode experiments, J. M. and K. S.-P. carried out the heart storage in transplantation medium experiments, T. K. supervised the mouse LAD experiments. M. R. F. and R. W. carried out the kinetic studies. R. C. H. supervised the chemical synthesis and helped direct the project and write the manuscript. M. P. M. and R. C. H. directed the project and wrote the manuscript, with assistance from all other authors.

## Supplementary Material

Supplemental Data

## Footnotes

4 The abbreviations used are:

SQR: sulfide:quinone oxidoreductase
PTM: posttranslational modification
NAP: *N*-acetylpenicillamine
IS: internal standards
TPP: triphenylphosphonium
LAD model: left anterior descending model
ACN: acetonitrile
FA: formic acid.
