# Supplementary material for: Assessment of H2S in vivo using the newly developed mitochondria-targeted mass spectrometry probe MitoA
Source: J Biol Chem. 2017 Mar 20;292(19):7761–73. doi: 10.1074/jbc.M117.784678 (PMC5427258; doi:10.1074/jbc.M117.784678)
Supplement: Supplemental Data [file supp_292_19_7761__index.html]

Assessment of H2S in vivo Using the Newly Developed Mitochondria-Targeted Mass Spectrometry Probe MitoA — Assessment of H2S in vivo using the newly developed mitochondria-targeted mass spectrometry probe MitoA — Measuring H2S with MitoA — Supplemental Data 

# Assessment of H2S *in vivo* using the newly developed mitochondria-targeted mass spectrometry probe MitoA

## Supplemental Data

- Supplemental Data (.pdf, 4.2 MB)
