# Supplementary material for: Assessment of H2S in vivo using the newly developed mitochondria-targeted mass spectrometry probe MitoA
Source: J Biol Chem. 2017 Mar 20;292(19):7761–73. doi: 10.1074/jbc.M117.784678 (PMC5427258; doi:10.1074/jbc.M117.784678)
Supplement: Supplemental Data [file 10.1074_M117.784678_jbc.M117.784678-1.pdf]

Supplementary Information

Assessment of H_2S *in vivo* using the recently developed mitochondria-targeted mass spectrometry probe MitoA

Sabine Arndt[a], Carlos D. Baeza-Garza[b], Angela Logan[a], Tiziana Rosa[c], Rudolf Wedmann[d], Tracy A. Prime[a], Jack L. Martin[e], Kourosh Saeb-Parsey[e], Thomas Krieg[c], *Milos*. R. Filipovic[d], Richard C. Hartley[b,*], and Michael P. Murphy[a,*]

[a]MRC Mitochondrial Biology Unit, Hills Road, Cambridge CB2 0XY, UK.
[b]WestCHEM School of Chemistry, University of Glasgow, Glasgow G12 8QQ, UK.
[c]Department of Medicine, University of Cambridge, Addenbrooke's Hospital, Hills Road, Cambridge, CB2 2QQ, UK.
[d]University of Bordeaux, IBGC, UMR 5095, F-33077 Bordeaux, France; 3 CNRS, IBGC, UMR 5095, F-33077 Bordeaux, France
[e]Department of Surgery and Cambridge NIHR Biomedical Research Centre, Biomedical Campus, University of Cambridge, Addenbrooke's Hospital, Hills Road, Cambridge, CB2 2QQ, UK.

A

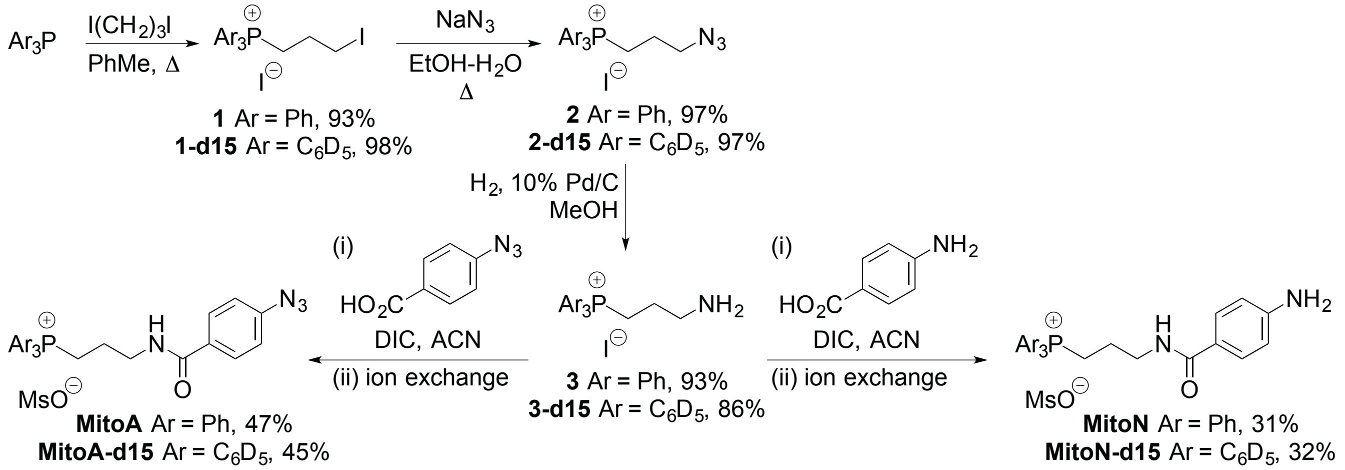

B

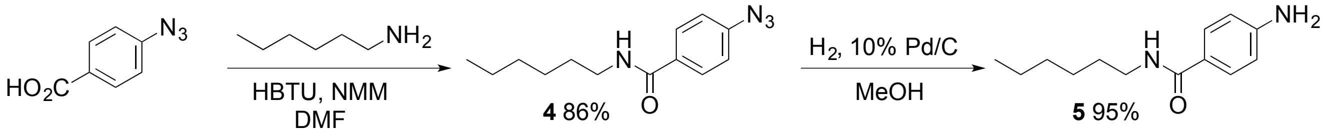

Fig. S1 (A) Synthesis of MitoA and MitoN. ACN = acetonitrile, DIC = *N, N'*-diisopropylcarbodiimide. (B) Control compounds **4** and **5** lacking the TPP targeting group were also prepared.Coupling of hexylamine with 4-azidobenzoic acid was achieved using *N,N,N',N'*-tetramethyl-*O*-(1*H*-benzotriazol-1-yl)uronium hexafluorophosphate (HBTU) to give amide **4**, which was then hydrogenated to give the amine **5**.

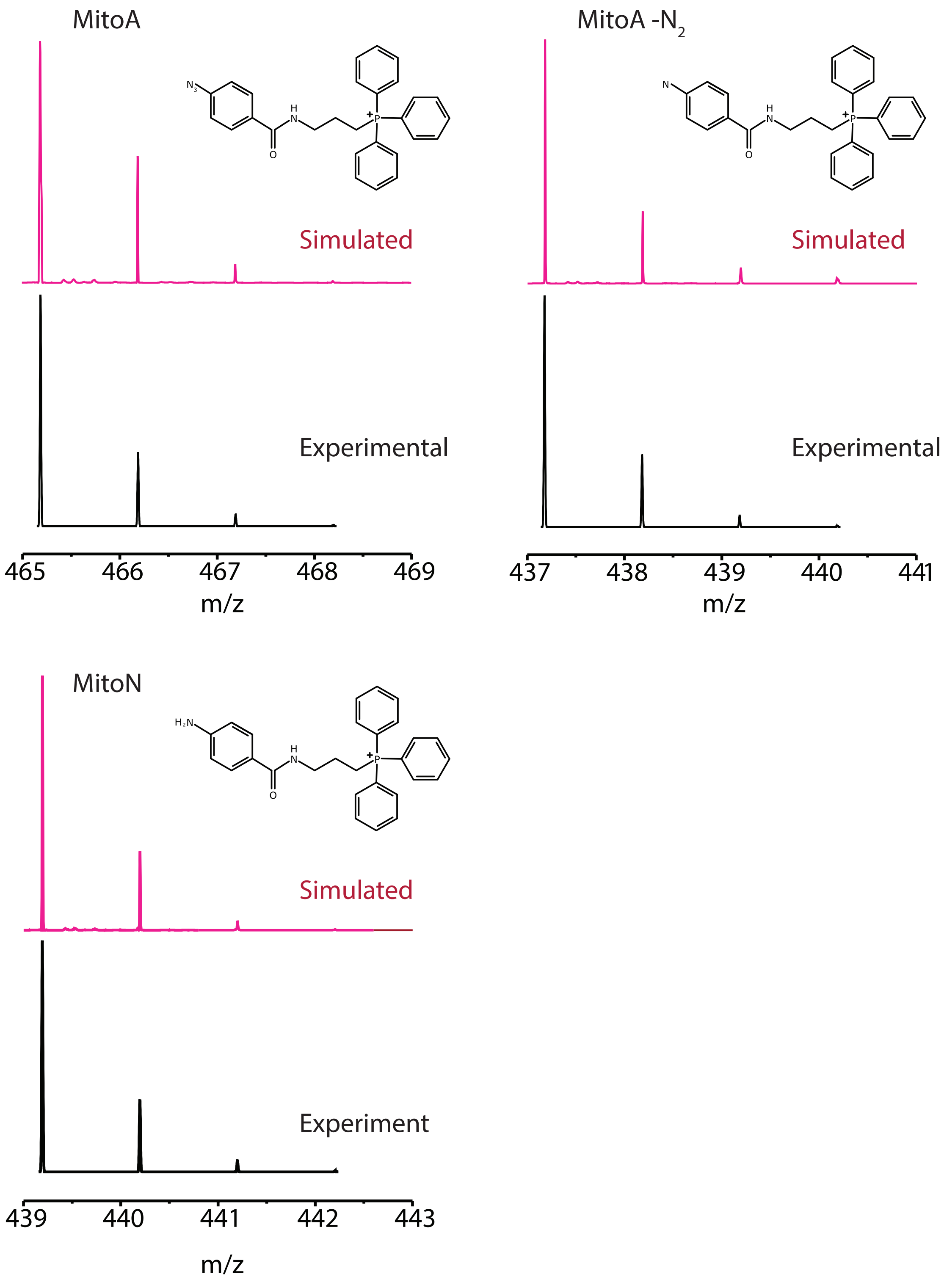

Fig. S2 Mass spectra of MitoA and MitoN. Observed (black) and simulated peaks (pink) for: MitoA, $[C_{28}H_{26}N_4OP]^+$ predicted m/z 465.1839. MitoA nitrene decomposition product from N_2 neutral loss $[C_{28}H_{26}N_2OP]^+$ predicted m/z 437.1782 MitoN, $[C_{28}H_{29}N_2OP]^+$ predicted m/z 439.1934.Spectra were obtained using an ultra-high resolution electron spray time of flight mass spectrometer (maXis Bruker Daltonics).

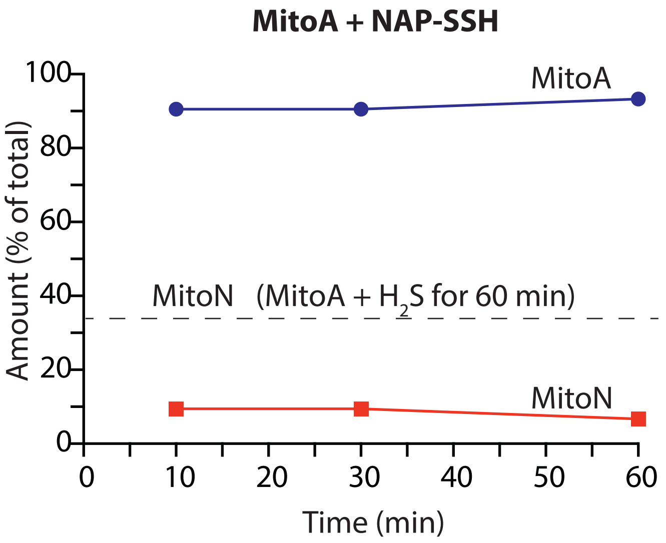

Fig. S3 Reactivity of MitoA with a persulfide. MitoA (10 µM) was mixed with NAP-SSH (25 µM) in KCl buffer, pH 7.2 at 37 °C. Samples were taken at the indicated times and analysed by RP-HPLC. The amounts of MitoA and MitoN were determined by RP-HPLC from standard curves and used to calculate the amount of that compound as a percentage of the sum of MitoA and MitoN. The dashed line shows the amount of MitoN formed by incubating MitoA (10 µM) NaS_2 (25 µM) in KCl buffer, pH 7.2 at 37 °C for 60 min.

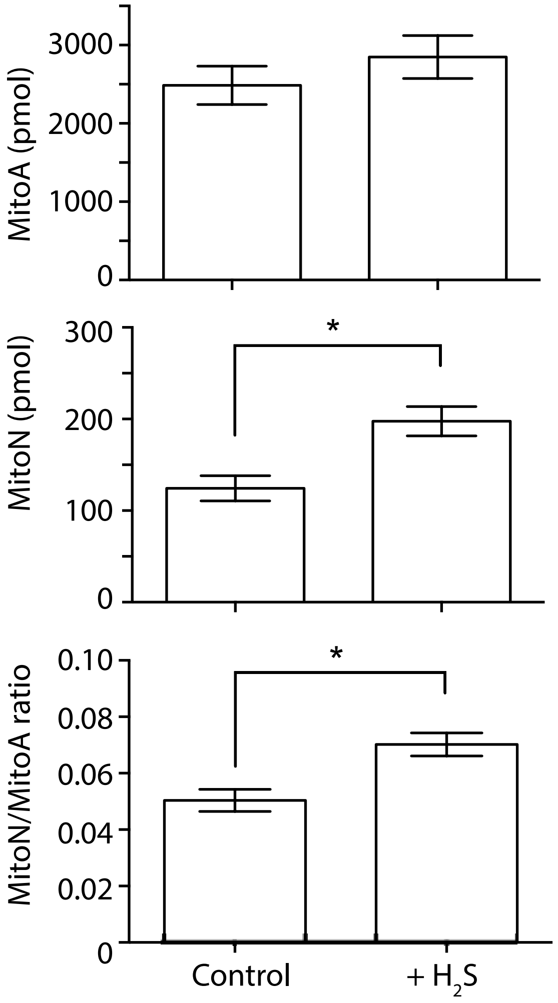

Fig. S4 Reactivity of MitoA with H_2S during extraction for mass spectrometry
MitoA (10 µM) was incubated with NaHS (10 µM) for 4 h in 95 % ACN. After the incubation, samples were dried under vacuum, resuspended in 20 % ACN, 0.1 % FA with internal standards and the amounts of MitoA, MitoN and the MitoN/MitoA ratio were quantified by LC-MS/MS. Data are means ± SEM, n = 4. *$p < 0.05$; two-tailed t-test.

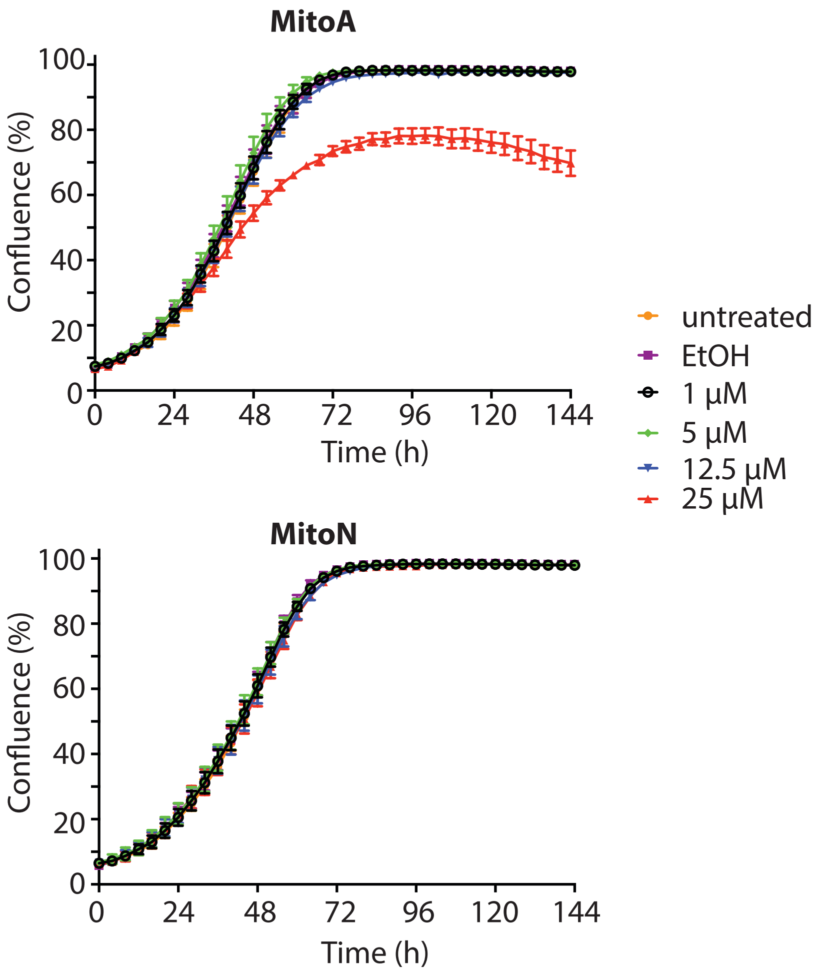

Fig. S5 Effect of MitoA and MitoN on cell proliferation. C2C12 cells were plated at 10,000 cells per well in 6-well plates (~1 000 cells per cm^2) and treating with MitoA or MitoN.Cell proliferation was then monitored by measuring confluency via automated bright field imaging (IncuCyte HD, EssenBioScience) for 144 h. Data are means ± SEM, n = 3.

Supplementary Experimental Procedures

Chemical syntheses

All reactions under an inert atmosphere were carried out using oven-dried or flame-dried glassware and solvents were added via syringe. Reagents were obtained from commercial suppliers and used without further purification. Dry solvents were collected from a Puresolv solvent purification system, obtained from commercial suppliers or dried in the laboratory. Ethanol was distilled from Mg turnings activated with iodine. ^{1}H NMR spectra were obtained using Bruker DPX/400 and Bruker-Avance III spectrometers operating at 400 and 500 MHz, ^{13}C NMR spectra at 101 and 126 MHz respectively. Signal splitting patterns were described as: singlet (s), doublet (d), triplet (t), quartet (q), multiplet (m), broad singlet (s, broad), or any combination of the above. All coupling constants were recorded in Hz. DEPT was used to assign the signals in ^{13}C NMR spectra as C, CH, CH_2 and CH_3.

(3-Iodopropyl)triphenylphosphonium iodide (**1**)

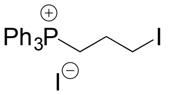

1,3-Diiodopropane (2.65 mL, 23 mmol) was added to a stirred solution of triphenylphosphine (3.01 g, 11.5 mmol) in toluene (25 mL) at 95 °C and the mixture was stirred for 20 h under reflux. After cooling to RT, the precipitate was collected by filtration and washed with Et_2O, after this, it was dried under reduced pressure to yield the alkyl iodide **1** as a white amorphous solid. (5.98 g, 93%). Mp: 172-174 °C. ν_{max} (ATR): 3056 (CH), 2916 (CH), 2849 (CH), 1587 (Ar). δ_H ($CDCl_3$, 400 MHz): 7.81-7.76 (9H, m, 6 *o*-H PPh_3, 3 *p*-H PPh_3), 7.71-7.66 (6H, m, 6 *m*-H PPh_3), 3.92-3.84 (2H, m, P-CH_2), 3.56 (2H, t, J = 6.4 Hz, I-CH_2), 2.19-2.10 (2H, m, CH_2-CH_2-CH_2). δ_C ($CDCl_3$, 125 MHz): 135.61 (d, J = 2.8 Hz, CH), 134.00 (d, J = 10.1 Hz, CH), 130.93 (d, J = 12.8 Hz, CH), 118.01 (d, J = 86.1 Hz, C), 27.08 (d, J = 1.8 Hz, CH_2), 24.41 (d, J = 51.3 Hz, CH_2), 6.96 (d, J = 20.1 Hz, CH_2). LRMS (ESI^+) 431 [M^+ (phosphonium cation), 100%]. HRMS (ESI^+): 430.9137. $C_{21}H_{21}IP$ requires M^+ (phosphonium cation), 430.9117.

(3-Iodoprop-1-yl)-tri(pentadeuterophenyl)phosphonium iodide (***d₁₅*-1**)

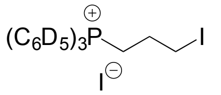

1,3-Diiodopropane (500 µL, 4.3 mmol) was added to a stirring solution of d-15 triphenylphosphine (500 mg, 1.8 mmol) in toluene (10 mL) at 95 °C and the mixture was set to react for 20 h under reflux conditions. After cooling to RT, the precipitate was collected by filtration and washed with Et_2O, after this, it was dried under reduced pressure to yield the alkyl iodide d_{15}-**1** as a white amorphous solid. (1.01 g, 98%). Mp: 173-175 °C. ν_{max} (ATR): 2859 (CH), 2343 (CD), 1559 (Ar). δ_H ($CDCl_3$, 500 MHz): 3.97-3.91 (2H, m, P-CH_2), 3.60 (2H, t, J = 6.5, I-CH_2), 2.21-2.14 (2H, m, CH_2-CH_2-CH_2). δ_C ($CDCl_3$, 125 MHz): 135.11-134.73 (m, CD), 133.65-133.16 (m, CD), 130.50-129.99 (m, CD), 117.2 (d, J = 86.2 Hz, C), 26.90 (d, J = 2.0 Hz, CH_2), 24.24 (d, J = 51.2 Hz, CH_2), 6.77 (d, J = 20.0 Hz, CH_2). LRMS (ESI^+) 446 [M^+ (phosphonium cation), 100%] HRMS (ESI^+): 446.1351. $C_{21}H_6D_{15}IP$ requires M^+ (phosphonium cation), 446.1362.

(3-Azidoprop-1-yl)triphenylphosphonium iodide (**2**)

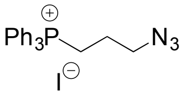

A solution of (3-iodoprop-1-yl)triphenylphosphonium iodide **1** (5.02 g, 9.0 mmol) and NaN_3 (1.17 g, 18.0 mmol) in EtOH (15.0 mL) and H_2O (15.0 mL) was stirred and heated to 70 °C for 24 h. After cooling, an extraction was carried out with $CHCl_3$ (3 30 mL). The combined organic layers were dried over $MgSO_4$, filtered and concentrated under reduced pressure to yield the alkyl azide **2** as an amorphous beige solid (4.13 g, 97%). MP: 151-153 °C δ_H ($CDCl_3$, 400 MHz): 7.85-7.79 (9H, m, 6 *o*-H PPh_3, 3 *p*-H PPh_3), 7.73-7.68 (6H, m, 6 *m*-H PPh_3), 3.95-3.87 (2H, m, P-CH_2), 3.82 (2H, t, *J* = 6.2 Hz, N_3-CH_2), 1.95-1.86 (2H, m, CH_2-*CH_2*-CH_2). δ_C ($CDCl_3$, 100 MHz): 135.57 (d, *J* = 2.9 Hz, CH), 133.95 (d, *J* = 10.3 Hz, CH), 130.92 (d, *J* = 12.5 Hz, CH), 118.05 (d, *J* = 86.6 Hz, C), 50.98 (d, *J* = 17.6 Hz, CH_2), 22.92 (d, *J* = 2.9 Hz, CH_2), 20.70 (d, *J* = 52.8 Hz, CH_2). ^{1}H and ^{13}C NMR data agree with those for the bromide salt [1].

(3-Azidoprop-1-yl)-tri(pentadeuterophenyl)phosphonium iodide (***d_{15}*-2**)

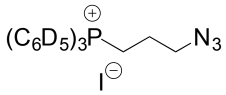

A solution of alkyl iodide ***d_{15}*-1** (1.00 g, 1.74 mmol) and NaN_3 (260 mg, 4.0 mmol) in EtOH (5.0 mL) and H_2O (5.0 mL) was stirred and heated to 70 °C for 24 h. After cooling, an extraction was carried out with DCM (3 30 mL). The combined organic layers were dried over $MgSO_4$, filtered and concentrated under reduced pressure to yield the alkyl azide ***d_{15}*-2** as an amorphous beige solid (825 mg, 97%). Mp: 151-153 °C. ν_{max} (ATR): 2888 (CH), 2796 (CH), 2362 (CD), 2110 (N_3), 1546 (Ar). δ_H ($CDCl_3$, 500 MHz): 3.83-3.76 (4H, m, P-CH_2, N_3-CH_2), 1.91-1.83 (2H, m, CH_2-*CH_2*-CH_2). δ_C ($CDCl_3$, 125 MHz): 134.99-134.60 (m, CD), 133.47-132.98 (m, CD), 130.40-129.91 (m, CD), 117.40 (d, *J* = 86.2 Hz, C), 50.71 (d, *J* = 17.5 Hz, CH_2), 22.63 (d, *J* = 2.6 Hz, CH_2), 20.33 (d, *J* = 52.9 Hz, CH_2). LRMS (ESI^+) 361 [M^+ (phosphonium cation), 34%], 333 [M^+ (phosphonium cation) – N_2, 100%]. HRMS (ESI^+): 361.2395. $C_{21}H_6D_{15}N_3P$ requires M^+ (phosphonium cation), 361.2409.

(3-Aminoprop-1-yl)triphenylphosphonium iodide (**3**)

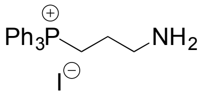

A solution of alkyl azide **2** (4.10 g, 8.7 mmol) and 10% Pd/C (425 mg, 4.0 mmol) in MeOH (40 mL) was stirred at RT under a H_2 (1 atm) atmosphere for 6 h. After this time, the mixture was filtered through Celite®. Then, the mixture was concentrated under reduced pressure to yield the amine **3** as an amorphous beige solid (3.62 g, 93%). Mp: 212-214 °C. ν_{max} (ATR): 3050 (NH), 2887 (CH), 2789 (CH), 1586 (Ar). δ_H ($CDCl_3$, 400 MHz): 7.83-7.76 (9H, m, 6 *o*-H PPh_3, 3 *p*-H PPh_3), 7.71-7.66 (6H, m, 6 *m*-H PPh_3), 3.78-3.71 (2H, m, P-CH_2), 3.01 (2H, t, *J* = 6.2 Hz, NH_2-*CH_2*), 1.88 (2H, s, NH_2), 1.82-1.72 (2H, m, CH_2-*CH_2*-CH_2). δ_C ($CDCl_3$, 100 MHz): 135.38 (d, *J* = 2.9 Hz, CH), 133.99 (d, *J* = 9.7 Hz, CH), 130.84 (d, *J* = 12.6 Hz, CH), 118.48 (d, *J* = 85.5 Hz, C), 41.74 (d, *J* = 17.5 Hz, CH_2), 26.13 (d, *J* = 4.9 Hz, CH_2), 21.14 (d, *J* = 51.5 Hz,

CH_2). LRMS (ESI^+) 320 [M^+ (phosphonium cation), 100%], HRMS (ESI^+): 320.1553. $C_{21}H_{23}NP$ requires M^+ (phosphonium cation), 320.1563.

(3-Aminopropyl)-tri(pentadeuterophenyl)phosphonium iodide (*d_{15}*-**3**)

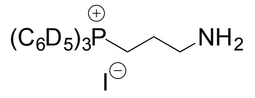

A solution of alkyl azide *d_{15}*-**2** (820 mg, 1.68 mmol) and 10% Pd/C (100 mg, 0.94 mmol) in MeOH (10 mL) was stirred at RT under a H_2 (1 atm) atmosphere for 6 h. After this time, the mixture was filtered through Celite®. Then, the mixture was concentrated under reduced pressure to yield the amine *d_{15}*-**3** as an amorphous beige solid (671 mg, 86%). Mp: 211-213 °C. ν_{max} (ATR): 2950 (NH), 2887 (CH), 2789 (CH), 2251 (CD), 1547 (Ar). δ_H ($CDCl_3$, 400 MHz): 3.79-3.72 (2H, m, P-CH_2), 3.01 (2H, t, J = 6.3 Hz, NH_2-*CH_2*), 1.80-1.71 (2H, m, CH_2-*CH_2*-CH_2), 1.48 (2H, s, NH_2). δ_C ($CDCl_3$, 100 MHz): 135.04-134.48 (m, CD), 133.70-133.09 (m, CD), 130.50-129.84 (m, CD), 118.15 (d, J = 85.7 Hz, C), 41.65 (d, J = 17.5 Hz, CH_2), 26.18 (d, J = 2.5 Hz, CH_2), 21.01 (d, J = 52.7 Hz, CH_2). LRMS (ESI^+) 335 [M^+ (phosphonium cation), 100%]. HRMS (ESI^+): 335.2489. $C_{21}H_8D_{15}NP$ requires M^+ (phosphonum cation), 335.2504.

[3-(4'-Azidobenzamido)-prop-1-yl]triphenylphosphonium mesylate (**MitoA**, mesylate salt)

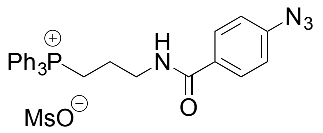

A solution of **3** (2.24 g, 5.0 mmol), 4-azidobenzoic acid (816 mg, 5.0 mmol), and *N, N'*-diisopropylcarbodiimide (800 µl, 5.2 mmol) in dry ACN (20 mL) was stirred at RT under argon for 48 h. After this, the precipitate was washed with ACN (5 × 10 mL). The precipitate was dried under high vacuum to remove the solvent, resulting in a brown powder. The powder was then treated for ion exchange with ion exchange resin Araldite® IRA-400 charged with mesylate ions giving a brown amorphous solid after solvent was removed. Column chromatography [SiO_2, DCM-MeOH (95:5)] yielded the mesylate salt of **MitoA** as a brown amorphous solid (1.34 g, 47%). R_f [SiO_2, DCM-MeOH (95:5)]: 0.25. Mp: 157-159 °C. ν_{max} (ATR): 3289 (NH), 3063 (CH), 2928 (CH), 2901 (CH), 2120 (N_3), 1653 (C=O), 1601 (Ar), 1530 (Ar), 1497 (Ar) cm^{-1}. δ_H ($CDCl_3$, 400 MHz): 9.28 (t, J = 5.9 Hz, NH), 8.15 (2H, d, J = 8.9 Hz, H-2', H-6'), 7.76-7.69 (9H, m, 3 × *p*-H PPh_3, 6 x *o*-H PPh_3), 7.64 – 7.59 (6H, m, 6 *m*-H PPh_3), 7.07 (2H, d, J = 8.8 Hz, H-3', H-5'), 3.75-3.67 (4H, m, P-*CH_2*, NH-*CH_2*), 2.81 (3H, s, *$CH_3SO_3^-$*), 1.98-1.90 (2H, m, CH_2-*CH_2*-CH_2). δ_C ($CDCl_3$, 100 MHz): 167.10 (C), 142.99 (C), 135.13 (d, J = 2.0 Hz, CH), 133.57 (d, J = 9.1 Hz, CH), 130.91 (C), 130.61 (d, J = 12.0 Hz, CH), 129.81 (CH), 118.92 (CH), 118.56 (d, J = 86.0 Hz, C), 39.78 (CH_3), 39.42 (d, J = 17.0 Hz, CH_2), 22.60 (d, J = 4.0 Hz, CH_2), 20.16 (d, J = 52.1 Hz, CH_2). LRMS (ESI^+) 465 [M^+ (phosphonium cation), 32%], 437 [M^+ (phosphonium cation) – N_2, 100]. HRMS (ESI^+): 465.1821. $C_{28}H_{26}N_4OP$ requires M^+ (phosphonum cation), 465.1839

[3-(4'-Azidobenzamido)propyl]tri(pentadeuterophenyl)phosphonium mesylate (***d_{15}*-MitoA** mesylate salt)

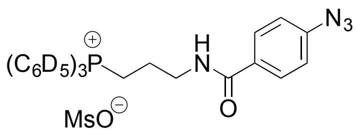

A solution of amine ***d_{15}*-3** (100 mg, 0.22 mmol), 4-azidobenzoic acid (36 mg, 0.22 mmol), and *N, N'*-diisopropylcarbodiimide (150 µl, 0.98 mmol) in dry ACN (2.5 mL) was stirred at RT under argon for 37 h. After this, the precipitate was washed with ACN (4 × 10 mL). The precipitate was dried under high vacuum to remove the solvent, resulting in a brown powder. The powder was then treated for ion exchange with ion exchange resin Araldite® IRA-400 charged with mesylate ions giving a brown amorphous solid after solvent was removed. Column chromatography [SiO_2, DCM-MeOH (95:5)] yielded the target compound as a brown amorphous solid (57 mg). As the mesylate ion was under integrated in the NMR, the solid was treated with aqueous $NaMeSO_3$ (0.5 M) and extracted with DCM. The DCM was removed under reduced pressure to give the mesylate salt of ***d_{15}*-MitoA** as a hygroscopic thick yellow oil (55 mg, 45%). R_f[SiO_2, DCM-MeOH (95:5)]: 0.27. Mp: 156-158 °C. ν_{max} (ATR): 3283 (NH), 2935 (CH), 2906 (CH), 2823 (CH), 2269 (CD), 2120 (N_3), 1644 (C=O), 1603 (Ar), 1546 (Ar), 1500 (Ar). δ_H ($CDCl_3$, 400 MHz): 9.10-9.08 (1H, m, NH), 7.98 (2H, d, J = 8.6 Hz, H-2', H-6'), 6.86 (2H, d, J = 8.6 Hz, H-3', H-5'), 3.53-3.46 (4H, m, P-*CH_2*, NH-*CH_2*), 2.57 (3H, s, *$CH_3SO_3^-$*), 1.83-1.81 (2H, m, CH_2-*CH_2*-CH_2). δ ($CDCl_3$, 100 MHz): 166.40 (C), 142.34 (C), 134.51-134.04 (m, CD), 132.96-132.36 (m, CD), 130.41 (C), 130.01-129.41 (m, CD), 129.31 (CH), 118.40 (CH), 117.66 (d, J = 86.1 Hz, C), 39.29 (CH_3), 39.06 (d, J = 17.0 Hz, CH_2), 22.14 (d, J = 4.1 Hz, CH_2), 19.66 (d, J = 52.3 Hz, CH_2). LRMS (ESI^+) 480 [M^+ (phosphonium cation), 30%], 452 [M^+ (phosphonium cation) – N_2, 100]. HRMS (ESI^+): 480.2760. $C_{28}H_{11}D_{15}N_4OP^+$ requires M^+ (phosphonum cation), 480.2780

[3-(4'-Aminobenzamido)prop-1-yl]triphenylphosphonium mesylate (**MitoN** mesylate salt).

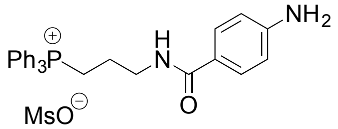

A solution of amine **3** (1.12 g, 2.50 mmol), 4-aminobenzoic acid (340 mg, 2.47 mmol), and *N, N'*-diisopropylcarbodiimide (400 µl, 2.68 mmol) in dry ACN (10 mL) was stirred at RT under argon for 48 h. After this, the precipitate was washed with ACN (5 × 10 mL). The precipitate was dried under high vacuum to remove the solvent, resulting in a beige powder. The powder was then treated for ion exchange with ion exchange resin Araldite® IRA-400 charged with mesylate ions giving a beige amorphous solid after solvent was removed. Column chromatography [SiO_2, DCM-MeOH (9:1)] yielded the mesylate salt of **MitoN** as a beige amorphous solid (410 mg, 31%). R_f[SiO_2, DCM-MeOH (9:1)]: 0.25. Mp: 206-208 °C. ν_{max} (ATR): 3333 (NH), 3225 (CH), 3059 (CH), 2931 (CH), 2904 (CH), 1628 (CO), 1603 (Ar), 1545 (Ar), 1508 (Ar). δ_H ($CDCl_3$, 400 MHz): 8.92 (t, J = 5.9 Hz, NH), 7.93 (2H, d, J = 8.6 Hz, H-2', H-6'), 7.76-7.68 (9H, m, 3 × *p*-H PPh_3, 6 × *o*-H PPh_3), 7.63 – 7.59 (6H, m, 6 × *m*-H PPh_3), 6.66 (2H, d, J = 8.5 Hz, H-3', H-5'), 4.07 (2H, s, NH_2) 3.74-3.67 (4H, m, P-*CH_2*, NH-*CH_2*), 2.83 (3H, s, *$CH_3SO_3^-$*), 1.97-1.88 (2H, m, CH_2-*CH_2*-

CH_2). δ_C ($CDCl_3$, 100 MHz): 167.74 (C), 149.59 (C), 134.92 (d, J = 2.3 Hz, CH), 133.41 (d, J = 9.0 Hz, CH), 130.42 (d, J = 12.5 Hz, CH), 129.47 (C), 123.71 (CH), 118.46 (d, J = 86.0 Hz, C), 114.07 (CH), 39.65 (CH_3), 39.06 (d, J = 17.0 Hz, CH_2), 22.64 (d, J = 3.0 Hz, CH_2), 19.79 (d, J = 52.0 Hz, CH_2). LRMS (ESI^+) 439 [M^+ (phosphonium cation), 100%]. HRMS (ESI^+): 439.1915. $C_{28}H_{28}N_2OP$ requires M^+ (phosphonum cation), 439.1934.

[3-(4'-Aminobenzamido)prop-1-yl]tri(pentadeuterophenyl)phosphonium mesylate (*d_{15}*-**MitoN** mesylate salt)

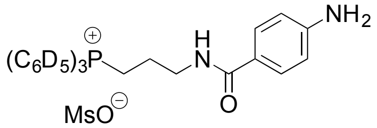

A solution of amine ***d_{15}*-3** (100 mg, 0.22 mmol), 4-aminobenzoic acid (30 mg, 0.21 mmol), and *N, N'*-diisopropylcarbodiimide (150 µl, 0.98 mmol) in dry ACN (2.5 mL) was stirred at RT under argon for 48 h. After this, the precipitate was washed with ACN (5 × 10 mL). The precipitate was dried under high vacuum to remove the solvent, resulting in a beige powder. The powder was then treated for ion exchange with ion exchange resin Araldite® IRA-400 charged with mesylate ions giving a beige amorphous solid after solvent was removed. Column chromatography [SiO_2, DCM-MeOH (9:1)] yielded the amide ***d_{15}*-MitoN** as a beige amorphous solid (50 mg, 42%). As the mesylate ion was under integrated in the NMR, the solid was treated with aqueous $NaMeSO_3$ (0.5 M) and extracted with DCM. The DCM was removed under reduced pressure to give the mesylate salt of ***d_{15}*-MitoN** as a beige solid (38 mg, 32%). R_f[SiO_2, DCM-MeOH (9:1)]: 0.25. Mp: 207-209 °C. ν_{max} (ATR): 3335 (NH), 3228 (CH), 2931 (CH), 2902 (CH), 2281 (CD), 1623 (CO), 1599 (Ar), 1549 (Ar), 1509 (Ar). δ_H (CD_3OD, 400 MHz): 7.56 (2H, d, J = 8.8 Hz, H-2', H-6'), 6.62 (2H, d, J = 8.8 Hz, H-3', H-5'), 3.50-3.38 (4H, m, P-CH_2, NH-CH_2), 2.65 (3H, s, CH_3SO_3), 1.96-1.86 (2H, m, CH_2-CH_2-CH_2). δ_C CD_3OD, (100 MHz): 170.54 (C), 153.45 (C), 136.06-135.57 (m, CD), 134.64-134.05 (m, CD), 131.37-130.74 (m, CD), 130.02 (C), 122.54 (CH), 119.54 (d, J = 86.4 Hz, C), 114.65 (CH), 40.78 (d, J = 19.0 Hz, CH_2), 39.52 (CH_3), 23.99 (d, J = 3.7 Hz, CH_2), 20.68 (d, J = 53.0 Hz, CH_2). LRMS (ESI^+) 454 [M^+ (phosphonium cation), 100%]. HRMS (ESI): 454.2855. $C_{28}H_{13}D_{15}N_2OP$ requires M^+ (phosphonum cation), 454.2875

4'-Azido-N-(hex-1-yl)benzamide (**4**)

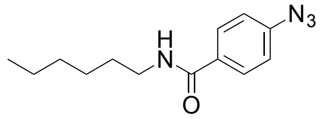

Hex-1-ylamine (320 µL, 2.4 mmol), HBTU (835 mg, 2.2 mmol), and *N*-methylmorpholine (NMM, 440 µL, 4 mmol) were added to a stirred solution of *para*-azidobenzoic acid (367 mg, 2.2 mmol) in anhydrous DMF (10 mL) at RT under argon. Stirring was continued for 18 h, and then water (15 mL) was added. The precipitate was collected by filtration and washed with water (3 × 10 mL). The resulting solid was dried under high vacuum to give the amide **4** as an amorphous yellow solid (427 mg, 86%). Mp: 71-73 °C. ν_{max} (ATR): 3312 (NH), 3075 (CH), 2955 (CH), 2928 (CH), 2860 (CH), 2137 (N_3), 1629 (CO), 1605 (Ar), 1545 (Ar), 1499 (Ar). δ_H ($CDCl_3$, 500 MHz): 7.76 (2H, d, J = 8.6 Hz, H-2', H-6'), 7.05 (2H, d, J = 8.6 Hz, H-3', H-5'), 6.14 (1H, s, NH), 3.45-3.41 (2H, m, CH_2-1), 1.60 (2H, qn, J = 7.0

Hz, CH_2-2), 1.40-1.28 (6H, m, CH_2-3, CH_2-4, and CH_2-5), 0.91-0.87 (3H, t, J = 7.0 Hz, CH_3). δ_C ($CDCl_3$, 125 MHz): 166.57 (C), 143.30 (C), 131.53 (C), 128.78 (CH), 119.03 (CH), 40.32 (CH_2), 31.65 (CH_2), 29.78 (CH_2), 26.82 (CH_2), 22.69 (CH_2), 14.16 (CH_3). LRMS (EI^+): 246 ($M^{+•}$, 47%), 218 ($M^{+•}$ – N_2, 100%), HRMS (EI^+): 246.1483. $C_{13}H_{18}N_4O$ requires $M^{+•}$, 246.1481.

4'-Amino-N-(hex-1-yl)benzamide (**5**)

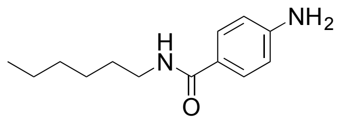

A solution of azide **4** (200 mg, 0.81 mmol) and 10% Pd/C (20 mg, 0.19 mmol) in MeOH (5 mL) was stirred at RT under a H_2 (1 atm) atmosphere for 3 h. After this time, the mixture was filtered through Celite®. Then, the mixture was concentrated under reduced pressure to yield amine **5** as an amorphous beige solid (168 mg, 95%). Mp: 102-104 °C. ν_{max} (ATR): 3410 (NH), 3329 (NH), 3215 (CH), 2955 (CH), 2924 (CH), 2855 (CH), 1637 (C=O), 1601 (Ar), 1537 (Ar), 1504 (Ar). δ_H ($CDCl_3$, 400 MHz): 7.58 (2H, d, J = 8.6 Hz, H-2', H-6'), 6.60 (2H, d, J = 8.6 Hz, H-3', H-5'), 6.26 (1H, broad s, NH), 4.05 (2H, s, NH_2) 3.45-3.41 (2H, m, CH_2-1]), 1.60 (2H, qn, J = 7.0 Hz, CH_2-2), 1.40-1.28 (6H, m, CH_2-3, CH_2-4 and CH_2-5), 0.91-0.87 (3H, t, J = 7.0 Hz, CH_3). δ_C ($CDCl_3$, 100 MHz): 166.57 (C), 152.43 (C), 133.65 (C), 128.88 (CH), 117.88 (CH), 40.27 (CH_2), 31.63 (CH_2), 29.75 (CH_2), 26.79 (CH_2), 22.67 (CH_2), 14.14 (CH_3). LRMS (ESI^+) 243 [$(M + Na)^+$, 100%] HRMS (ESI^+): 243.1469. $C_{13}H_{20}N_2ONa$ requires $(M + Na)^+$, 243.1468. IR data in broad agreement with Lit. [2].

Supplementary References

1. J. D. Knight, S. J. Sauer and D. M. Coltart, *Org. Lett.*, 2011, **13**, 3118-3121.
2. C. R. Clark, M. J. M. Wells, R. T. Sansom, G. N. Norris, R. C. Dockens and W. R. Ravis, *J. Med. Chem.*, 1984, **27**, 779-782.
